# Inhibitor design for TMPRSS2: insights from computational analysis of its backbone hydrogen bonds using a simple descriptor

**DOI:** 10.1007/s00249-023-01695-4

**Published:** 2023-12-29

**Authors:** Suraj Ugrani

**Affiliations:** Purdue University, West Lafayette, IN 47907 USA

**Keywords:** SARS-CoV-2, TMPRSS2, Protease inhibitors, Hydrogen bond wrapping, Molecular descriptor

## Abstract

Transmembrane protease serine 2 (TMPRSS2) is an important drug target due to its role in the infection mechanism of coronaviruses including SARS-CoV-2. Current understanding regarding the molecular mechanisms of known inhibitors and insights required for inhibitor design are limited. This study investigates the effect of inhibitor binding on the intramolecular backbone hydrogen bonds (BHBs) of TMPRSS2 using the concept of hydrogen bond wrapping, which is the phenomenon of stabilization of a hydrogen bond in a solvent environment as a result of being surrounded by non-polar groups. A molecular descriptor which quantifies the extent of wrapping around BHBs is introduced for this. First, virtual screening for TMPRSS2 inhibitors is performed by molecular docking using the program DOCK 6 with a Generalized Born surface area (GBSA) scoring function. The docking results are then analyzed using this descriptor and its relationship to the solvent-accessible surface area term ΔG_sa_ of the GBSA score is demonstrated with machine learning regression and principal component analysis. The effect of binding of the inhibitors camostat, nafamostat, and 4-guanidinobenzoic acid (GBA) on the wrapping of important BHBs in TMPRSS2 is also studied using molecular dynamics. For BHBs with a large increase in wrapping groups due to these inhibitors, the radial distribution function of water revealed that certain residues involved in these BHBs, like Gln438, Asp440, and Ser441, undergo preferential desolvation. The findings offer valuable insights into the mechanisms of these inhibitors and may prove useful in the design of new inhibitors.

**Supplementary Information:**

The online version contains supplementary material available at 10.1007/s00249-023-01695-4.

## Introduction

Structure-based in silico methods are ubiquitous in drug discovery (Gorostiola González et al. 2022; Gupta et al. 2023; Opo et al. 2021; Sabe et al. 2021; Wang et al. 2020). Techniques like molecular dynamics (MD) simulation allow us to design new drug molecules by studying protein–ligand interactions of existing drugs or to screen a library of potential lead compounds by predicting their binding affinity to the target protein (Adelusi et al. 2022; Varela-Rial et al. 2022; Wu et al. 2022). However, techniques with higher accuracy are computation intensive. For instance, the cost of calculating non-bonded interactions in an all-atom MD simulation scales by O(*n*^2^), where *n* is the total number of particles in the system (Jung et al. 2019). This makes simulating biological systems for any useful length of time prohibitively expensive. Hence, approximate techniques such as molecular docking, which can be used for binding affinity prediction, and pharmacophore modeling, which helps in identifying key protein–ligand interactions, play a crucial role in the initial stages of drug discovery (Blanes-Mira et al. 2022; Giordano et al. 2022; Varela-Rial et al. 2022). They are especially helpful in situations that require urgent response such as the recent COVID-19 pandemic.

The severe acute respiratory syndrome coronavirus 2 (SARS-Cov-2) which causes the respiratory infectious disease COVID-19 emerged in Wuhan, Hubei province, China, in December 2019 (Huang et al. 2020; Zhou et al. 2020). The ensuing rapid global spread and severity of the disease prompted the World Health Organization (WHO) to declare the disease a pandemic on March 11, 2020 (Cucinotta and Vanelli 2020). The magnitude of its impact on human health and virtually all life aspects urged the scientific and medical communities to develop treatments, resulting in the discovery of vaccines for the original SARS-Cov-2 in record time (Polack et al. 2020; Tanne 2020). The Pfizer-BioNTech (BNT162b2) and Oxford-AstraZeneca (ChAdOx1) vaccines were among the first to exhibit promising results in terms of safety and efficacy (Voysey et al. 2021; Wallace et al. 2021). Recently, a bivalent booster vaccine (mRNA-1273.214) containing messenger RNAs encoding the Omicron variant spike protein was developed by Moderna which shows a superior response compared to the original mRNA-1273 against Omicron (Chalkias et al. 2022).

Even with the effectiveness of vaccines, the situation continues to evolve rapidly with the frequent emergence of newer subvariants more resistant to vaccine-elicited antibodies (Chatterjee et al. 2023; Cox et al. 2023). This is because as the virus circulates among populations, mutations accumulate in viral proteins. These mutations may alter their epitopes, lowering the effectiveness of antibodies, or result in increased transmissibility due to stronger binding to host proteins. For instance, the SARS-CoV-2 Omicron variant is characterized by more than 30 mutations in the spike protein, with 15 of them occurring in the receptor binding domain (RBD) (Hoffmann et al. 2021b; Planas et al. 2022). The recent recombinant Omicron subvariant XBB.1.5 displays enhanced transmissibility and greater antibody evasion compared to its predecessors (Uriu et al. 2023; Yue et al. 2023). One possibility to circumvent this obstacle is the inhibition of host cell proteases that are employed by the virus for cell entry (Chitalia and Munawar 2020; Hoffmann et al. 2021a; Zabiegala et al. 2023).

The cell entry of SARS-CoV-2 involves several host proteins; receptors like angiotensin-converting enzyme 2 (ACE2), coreceptors like neuropilin-1, and cofactors like furin and transmembrane serine protease 2 (TMPRSS2) (Alipoor and Mirsaeidi 2022; Daly et al. 2020; Jackson et al. 2022). The entry may occur via either the endocytosis or the membrane fusion pathways, the details of which have been discussed in several articles (Hoffmann et al. 2020a, b; Jackson et al. 2022; Peng et al. 2021; Shang et al. 2020; Zabiegala et al. 2023). The viral spike protein (S) consists of sub-units S1 and S2, where S1 enables the virus to bind to ACE2 receptors on the host cell surface. In the membrane fusion pathway, host proteases such as TMPRSS2 prime the bound S by cleavage at sites S1/S2 followed by S2’. This then initiates the fusion of the S2 sub-unit with the host cell membrane and enables cell entry. While other proteases such as cathepsin B and L can also prime the spike protein, inhibiting TMPRSS2 in certain cell lines such as human lung cells proves highly effective in blocking SARS-CoV-2 infection, making it a valuable target (Hoffmann et al. 2020a, b; Peng et al. 2021). Additionally, TMPRSS2 is also implicated in the entry mechanism of other coronaviruses including SARS and MERS (Middle Eastern Respiratory Syndrome), making TMPRSS2 inhibitors potential broad-spectrum antivirals (Shen et al. 2017).

**Fig. 1:**
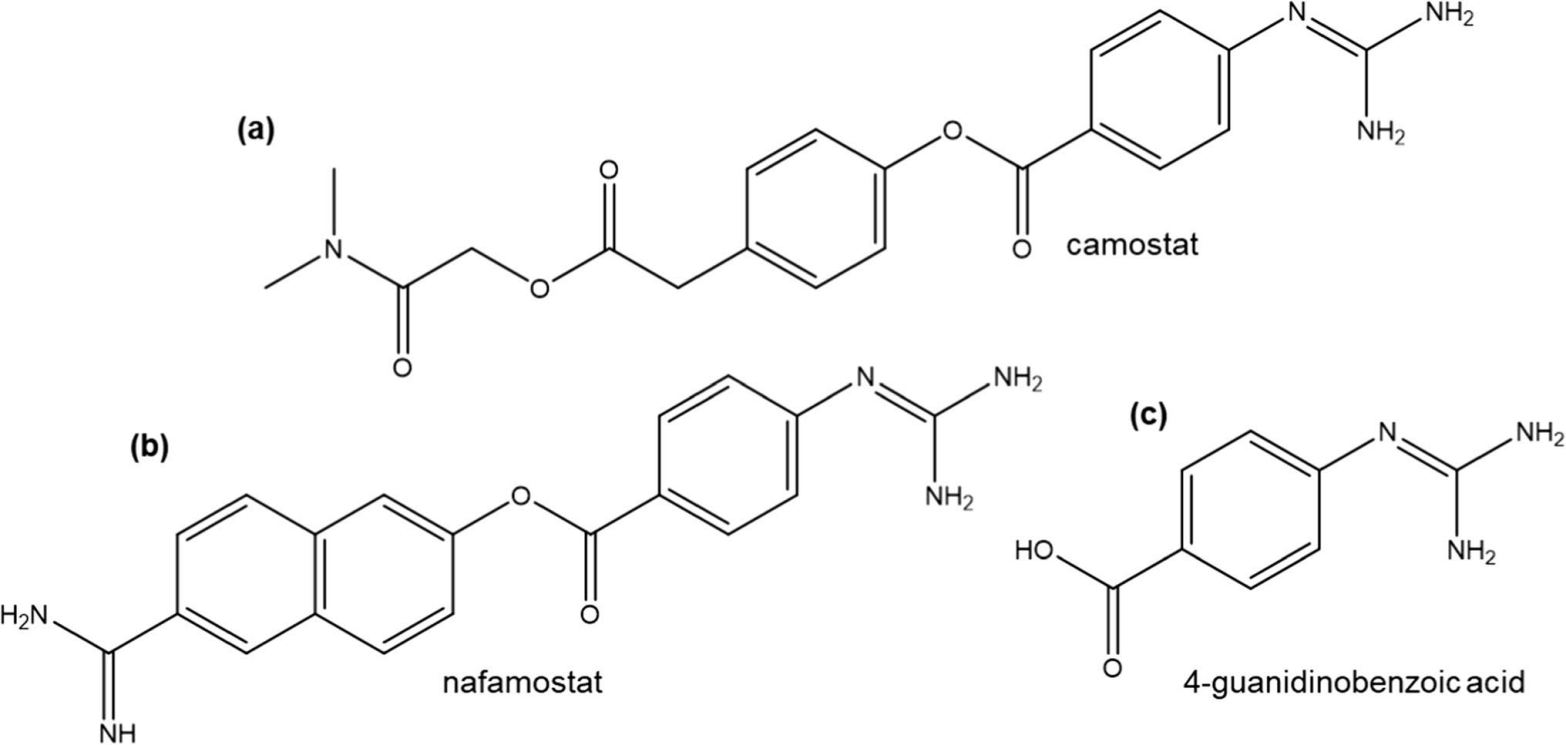
Two-dimensional structure of TMPRSS2 inhibitors **a** camostat, **b** nafamostat, and **c** 4-guanidinobenzoic acid

Despite its importance, relatively few approved drugs such as camostat and nafamostat have been demonstrably identified as TMPRSS2 inhibitors (Hoffmann et al. 2021a; Hoffmann et al. 2020a, b). Figure 1 shows the molecular structures of these inhibitors and their common metabolite 4-guanidinobenzoic acid (GBA). Additionally, strategies for the design of new inhibitors remain largely unexplored. While the inhibition mechanisms of these drugs and important interactions such as van der Waals and hydrogen bonding between inhibitors and TMPRSS2 have been examined recently in several virtual screening studies (Chikhale et al. 2020; Fraser et al. 2022; Hempel et al. 2021; Idris et al. 2020; Shakya et al. 2021; Sonawane et al. 2021; Tachoua et al. 2023), the focus of this work is the effect of inhibitor binding on the intramolecular backbone hydrogen bonds (BHBs) of TMPRSS2. Here, molecular docking and MD simulations are performed, followed by an analysis of the results to identify important intramolecular BHBs in TMPRSS2, which could be instrumental in inhibitor design.

## Theoretical background

### Wrapping of hydrogen bonds

It is known that the presence of nearby hydrophobic groups enhances dielectric-dependent pairwise interactions such as hydrogen bonds by shielding them from water (Bissantz et al. 2010; Fernández and Stephen Berry 2002; Grdadolnik et al. 2017). The concept of hydrogen bond wrapping in proteins describes the surrounding of electrostatic interactions like preformed amide-carbonyl backbone hydrogen bonds (BHBs) by hydrophobic groups, which results in desolvation of the region, thereby strengthening the interaction (Cramer et al. 2020; Fernández and Scott 2003a, b). It has been experimentally estimated in different studies that intramolecular hydrogen bonds in proteins may be up to 1–1.2 kcal/mol stronger in a well-wrapped hydrophobic microenvironment as opposed to being solvent-exposed (Fernández and Scott 2003a; Gao et al. 2009). Since the solvent environment of a protein significantly affects its intramolecular energy, the thermodynamic benefit of such water expulsion plays a key role in stabilizing the conformations of a protein and its association with ligands or other proteins (Dahanayake and Mitchell-Koch 2018; Fernández and Scott 2003b; Pietrosemoli et al. 2007; Pradhan et al. 2016).

The binding of an inhibitor results from favorable interactions of the protein’s polar and non-polar regions with the ligand’s corresponding complementary moieties. It also involves the displacement of water molecules surrounding insufficiently dehydrated BHBs located near the binding pocket (Chen et al. 2023; Fernández and Scheraga 2003). Hence, inhibitor design can be guided by knowledge of BHBs in the protein which are dehydration-sensitive i.e., have the greatest propensity for dehydration (Fernández 2005; Fernández et al. 2007; Irwin et al. 2019). Modifying a ligand such that it contributes one or more wrapping groups to BHBs that would gain the most stability on water removal can improve not only its binding affinity to the target (Cramer et al. 2020; Magarkar et al. 2019), but also its specificity (Fernández 2005). This has been demonstrated for the kinase inhibitor imatinib in a study where its specificity toward a particular target C-Kit was enhanced by such a modification (Fernández et al. 2007).

It has been shown that the number of non-polar groups wrapping a BHB can be used as an approximate measure of the extent of its dehydration (Fernández and Scott 2003a; Fernández and Stephen Berry 2002). However, identifying dehydration-sensitive BHBs is not trivial due to the presence of several interdependent interactions (Said and Hangauer 2015). With the help of molecular docking, machine learning, and molecular dynamics (MD) simulations, this work aims to identify and study dehydration-sensitive BHBs in TMPRSS2 using a simple descriptor. This descriptor is calculated for a BHB by merely counting the number of specific non-polar groups (carbonaceous groups CH_n_, with *n* = 1, 2, 3) within a ‘desolvation region’ surrounding it and is defined in the Methods section.

### MM-GBSA

MM-GBSA is an end-point implicit solvent method to estimate the free energy of binding for a protein–ligand (PL) complex. It applies molecular mechanics (MM) force fields to calculate bonded and non-bonded interactions, while the effect of solvent is calculated using a combination of the generalized Born (GB) model and the solvent accessible surface area (SA) model for electrostatic and non-electrostatic contributions, respectively (Genheden and Ryde 2015; Wang et al. 2019). The binding free energy is calculated by finding the difference in free energy between the PL complex, and the two uncomplexed species:

1 $${\Delta G}_{{\text{bind}}}= \langle {G}_{{\text{PL}}}\rangle -\langle {G}_{{\text{P}}}\rangle -\langle {G}_{{\text{L}}}\rangle$$

where the free energy *G* for any of the three species is decomposed into separate interactions as

2 $${\text{G}}={E}_{{\text{MM}}}+{G}_{{\text{GBSA}}}-TS$$

where the MM term *E*_MM_ comprises the sum of all bonded interactions like angle and dihedral energies (*E*_bnd_), as well as the electrostatic (*E*_el_) and van der Waals (*E*_vdW_) interactions in the gas phase. The solvation term *G*_GBSA_ is calculated as the sum of the electrostatic (*G*_gb_) and non-electrostatic (*G*_sa_) interactions due to solvent. *T* and *S* are the temperature and entropy, respectively. Hence,

3 $${\text{G}}={E}_{{\text{bnd}}}+{E}_{{\text{el}}}+{E}_{{\text{vdW}}}+{G}_{{\text{gb}}}+{G}_{{\text{sa}}}-TS$$

4 $$\therefore {\Delta G}_{{\text{bind}}}={\Delta E}_{{\text{bnd}}}+{\Delta E}_{{\text{el}}}+\Delta {E}_{{\text{vdW}}}+\Delta {G}_{{\text{gb}}}+{\Delta G}_{{\text{sa}}}-T\Delta {\text{S}}$$

Typically, Δ*G*_bind_ is calculated with the ensemble average of terms as denoted by <  > in Eq. (1) using frames from MD simulations of the protein, the ligand, and the complex. The current work utilizes the DOCK scoring function ‘Hawkins GB/SA’ for analysis, which is based on the GB solvation model proposed by Hawkins and co-workers (Hawkins et al. 1995, 1996). It assumes Δ*E*_bnd_ to be 0 and neglects the entropy term and is defined as follows:

5 $$\mathrm{Hawkins GB}/\mathrm{SA score}={\Delta E}_{{\text{el}}}+\Delta {E}_{{\text{vdW}}}+\Delta {G}_{{\text{gb}}}+{\Delta G}_{{\text{sa}}}$$

The Δ*G*_sa_ term accounts for the formation of the solute cavity and the short-range van der Waals forces and is associated with the solute’s interaction with the so-called first solvation shell (Hawkins et al. 1996; Wang et al. 2019). This term is taken to be proportional to the solute’s solvent-accessible surface area.

Since the wrapping of BHBs and the Δ*G*_sa_ term are both related to the stability of solute-adjacent solvent, we hypothesize that the effect of inhibitor binding on the solvent environment of BHBs could be studied using information about their wrapping. In the current study, this hypothesis is tested for the protein TMPRSS2 using molecular docking, machine learning, and MD simulation.

## Methods

### Ligands preparation

A list of 1174 small molecule protease inhibitors with molecular weights less than 1000 g/mol and greater than 100 g/mol was obtained from the MEROPS protease database (Rawlings et al. 2018). The molecules were limited to those containing C, H, O, N, S, Cl, Br, I, and F. The PubChem CID numbers and InChI format representations of the molecules were obtained with the help of PubChemPy. The 3D structures for these molecules were then generated with the RDKit module (https://www.rdkit.org) of Python using the MMFF94s force field. Using UCSF Chimera (Pettersen et al. 2004), the molecules were then assigned protonation states reasonable at physiological pH and Gasteiger–Marsili partial atomic charges, and finally subjected to energy minimization. A similar procedure was followed to prepare the inhibitors nafamostat and camostat, along with their metabolite 4-guanidinobenzoic acid (GBA), for docking.

### Receptor preparation

The crystal structure of the TMPRSS2 serine protease domain in its bioactive form was obtained from the Protein Data Bank (PDB ID: 7MEQ) (Fraser et al. 2022). The ligand and water molecules were removed and only atoms of the protein were retained. The residue Ser441 which is covalently bonded to the ligand was returned to its original form. The DockPrep tool in Chimera was then used to add hydrogens and partial atomic charges from the AMBER ff14SB force field and optimize the receptor.

### Preparation for molecular docking

The program UCSF DOCK 6 (Allen et al. 2015) was used for molecular docking. The following preprocessing was performed using Chimera and DOCK’s accessory programs: first, the molecular surface of the receptor without hydrogens was calculated using the ‘DMS’ tool available in Chimera. Next, the spheres which characterize the ligand binding site were generated using the accessory sphgen with a probe radius of 1.4 Å. Only spheres within 8 Å of the original ligand’s position were retained. A box was then created around the spheres maintaining a margin of 5 Å using the accessory program showbox. Finally, the scoring grid used to calculate the interaction between ligand and receptor was generated within the box with a resolution of 0.3 Å. The grid uses a 6–12 Lennard Jones potential for modeling the van der Waals interactions, with 6 and 12 being the attractive and repulsive exponents respectively, and a Coulombic potential for electrostatic interactions. DOCK’s default scoring function called the grid-based score is the sum of these two terms.

### Molecular docking

Docking was carried out in two steps. First, the molecules were docked to the receptor structure, allowing for ligand flexibility, and scored with DOCK’s grid-based scoring function using the generated grid. This initial docking was followed by a rescoring step using the more involved Hawkins GB/SA scoring function. Starting with the pose from the first docking step, a 1000-step energy minimization was performed to allow each ligand to reach a minima with respect to the new score. This involves the optimization of the ligand interactions by translating, rotating, and refinement of torsion angles. The total Hawkins GB/SA score was then calculated for each minimized ligand along with contributions of the individual terms including Δ*G*_sa_.

### Descriptor calculation

The descriptors or the ‘nonpolar wrapping groups count’ for the complex of a particular ligand with the receptor were calculated from its final docking pose. It is defined for a backbone hydrogen bond (BHB), as the number of non-polar groups (CH_*n*_, with *n* = 1, 2, 3) not attached to a polar atom in its surrounding desolvation region. This region is taken to be two spheres of radii 0.65 nm centered at the alpha-carbon of each residue involved in the BHB. This definition is similar to the one that appears in the original work (Fernández and Scheraga 2003). These were calculated in the molecular visualization program PyMol using the plugin ‘wrappy’ (Martin 2012; Warren 2002). To identify BHBs, the maximum allowed deviation from the optimal hydrogen bonding angle was taken as 40° and the donor–acceptor distance cutoff as 0.35 nm. The receptor was found to have 126 BHBs and hence each complex is characterized by 126 descriptors. Finally, the descriptor values for the uncomplexed receptor were subtracted from the corresponding descriptor values for all complexes so that the data describes the additional wrapping due to the binding of ligands.

### Machine learning

**Fig. 2:**
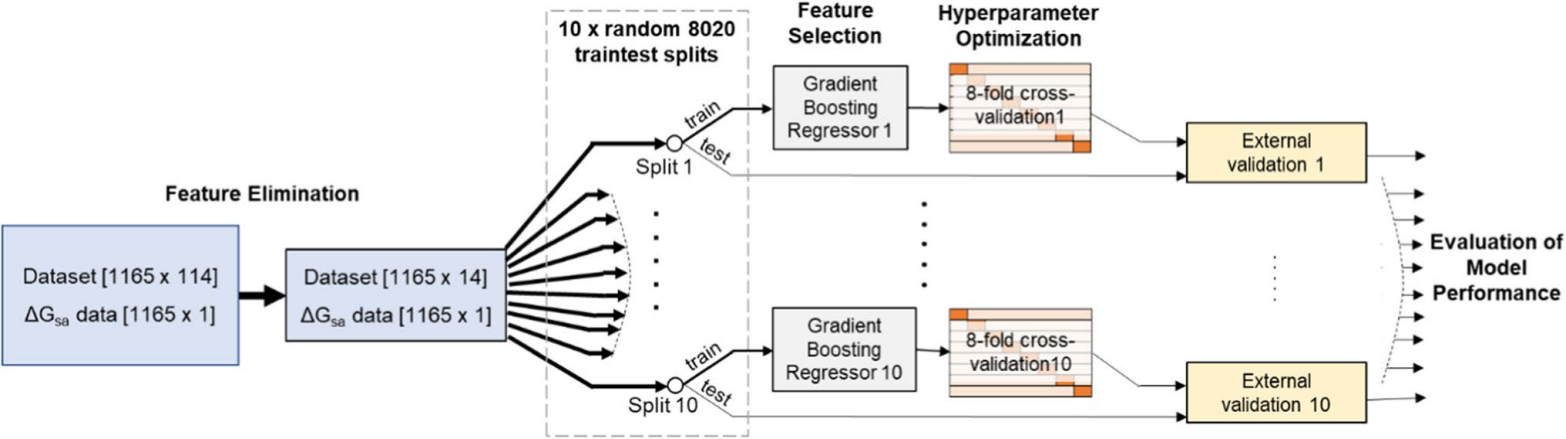
Schematic of the workflow for performing regression using machine learning algorithms

Regression with machine learning algorithms was used in the current work to test the hypothesis introduced earlier, which posits a relationship between the wrapping of BHBs and the surface area term Δ*G*_sa_. The schematic of the machine learning workflow is illustrated in Fig. 2. The regression models were created and analyzed using the Python library scikit-learn (Pedregosa et al. 2011). The dataset consisting of 126 wrapping descriptors and the surface area terms of 1165 complexes was first pre-processed. Features with very little or no variance (< 0.01) were eliminated. These mostly represent BHBs farther from the binding pocket since the presence of the bound ligand does not influence their wrapping count. Furthermore, if two features had a correlation coefficient of 0.7 or more, the feature with the lower variance was dropped. A residue pair having two BHBs between them was considered a single feature since the desolvation region as described above, and hence the descriptor value, is the same for any hydrogen bond between that pair.

At this time, the dataset was split into a training set consisting of 80% of the data and a test set with the remaining data. Feature selection was carried out with the training set based on feature importance computed using a gradient boosting regressor. Regression was then carried out using random forest regressors (RFRs), gradient boosting regressors (GBRs), support vector regressors (SVRs), and linear regression (LR). Hyperparameters for all machine learning algorithms were optimized by a grid search using the training data, with an eightfold cross-validation scheme. The performance of the models on the test set was then evaluated by calculating the Pearson correlation coefficient (PCC) and root mean squared error (RMSE) between the predictions of Δ*G*_sa_ and its values from docking. These models, trained on 80% of the dataset, were also used to predict the surface area terms for the three known inhibitors.

Furthermore, a principal component analysis (PCA) of the entire dataset was carried out to visualize the high-dimensional data and identify any underlying trends. The variance in the data explained by the first five principal components was also analyzed and loadings of the various features were compared to the feature importance values previously calculated.

### MD simulation

Camostat, nafamostat, and their common metabolite 4-guanidinobenzoic acid (GBA) are known covalent inhibitors of TMPRSS2 (Fraser et al. 2022; Hempel et al. 2021; Hoffmann et al. 2021a). To understand the wrapping patterns for these inhibitors, MD simulations were carried out for each of their non-covalent complexes which precede the formation of the covalent bond, and also for the apo TMPRSS2. This was done using GROMACS 2022.3 with the OPLS-AA force field (Hess et al. 2008). The ligand topologies were generated using the web server LigParGen (Dodda et al. 2017). The receptor from docking was used and the docked poses of the ligands were taken as the starting point for the complexes.

The receptor or its complex was first solvated with the TIP3P model of water in a dodecahedron box with periodic boundary conditions, followed by the addition of an appropriate number of Cl^−^ ions to bring the net charge of the system to 0. This system was first subjected to energy minimization by the steepest descent algorithm until the maximum force was less than 1000 kJ/(mol. nm). Two equilibration steps were then carried out under an NVT followed by an NPT ensemble, for 100 ps each. Finally, the equilibrated system was subjected to a production run of 180 ns. All simulations had a step size of 2 fs and were carried out at 300 K and 1 bar maintained using a modified Berendsen thermostat and a Parrinello–Rahman barostat, respectively. Data were collected at 10 ps intervals.

To ensure the stability of the protein and the simulation as a whole, the root mean square deviation (RMSD) of the protein backbone with reference to the starting structure, along with its radius of gyration (*R*_g_) and root mean square fluctuation (RMSF) were observed. The solvent accessible surface area (SASA) of the protein during the simulations was also plotted. The simulations were analyzed using data collected from the last 50 ns of each trajectory. To study the wrapping patterns of BHBs in the four cases, 1000 frames separated by 50 ps intervals were extracted from respective trajectories, and descriptors were calculated as described above for each frame. This data were then analyzed to infer the stability of these BHBs. The arrangement of solvent surrounding a BHB was observed by calculating the radial distribution function (RDF) of the oxygen atom of water around its hydrogen bond acceptor to identify BHBs that undergo desolvation due to the inhibitors.

## Results and discussion

### Molecular docking

Virtual screening for TMPRSS2 inhibitors was carried out with 1174 small molecules having molecular weights between 100 g/mol and 1000 g/mol from the MEROPS protease inhibitor database and the structure of TMPRSS2 from the Protein Data Bank. The preparation of the ligands and the receptor for molecular docking with DOCK was carried out using Chimera and DOCK’s accessory programs. Docking was first carried out using the default grid-based score. The scores for this initial docking ranged from − 83.58 kcal/mol to − 17.88 kcal/mol, with all negative values except for one. These poses were then subject to energy minimization by allowing translation, rotation, and refinement of torsion angles of the molecules, and finally rescored using the Hawkins GB/SA score. The negative scores for this step ranged from − 60.56 kcal/mol to − 1.73 kcal/mol. Nine compounds showed positive scores with few having unrealistically high values despite having negative grid-based scores, revealing unsuccessful energy minimization. This is likely caused by the difference between the two scoring functions. Since the GB/SA score considers more interactions than the grid-based score, the two will have different energy surfaces and the minima of one may not coincide with that of the other. These compounds were excluded from the following analyses to avoid using unrealistic conformations. The complete list of scores for both docking steps is given in Online Resource 1.

**Fig. 3:**
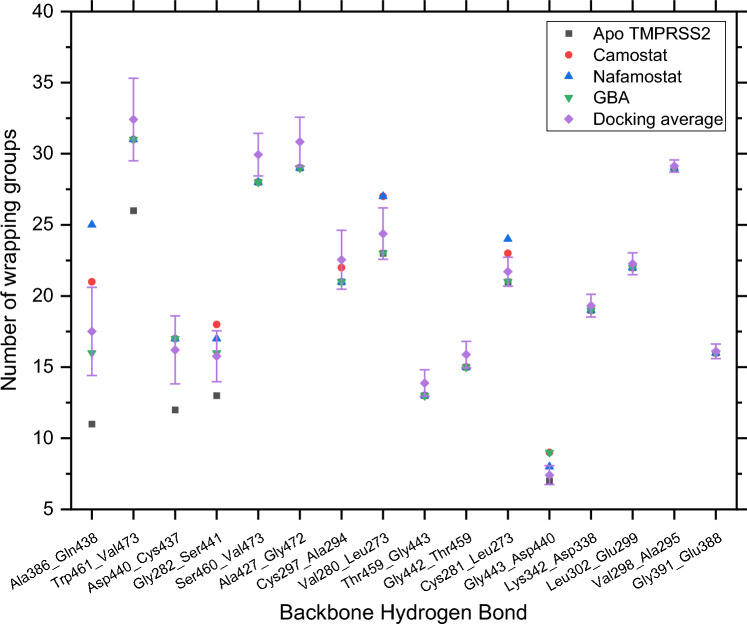
Docking Result—Number of wrapping groups for 16 BHBs in case of apo TMPRSS2, average number groups added for 1165 docked ligands, and groups added for docked inhibitors camostat, nafamostat, and GBA

The compounds with the ten best Hawkins GB/SA scores which are potential TMPRSS2 inhibitors are given in Table 1 along with their PubChem CIDs, both docking scores, and the residues with which they interact via hydrogen bonding, hydrophobic effect, or other interactions in their final pose. This was obtained using the Protein–Ligand Interaction Profiler web tool (Adasme et al. 2021). Their molecular structures are provided in Online Resource 2. The cumulative docking results with respect to the number of wrapping groups added (ΔGroups) for 16 backbone hydrogen bonds (BHBs) with the largest values are given in Table 2 and with respect to the total number of wrapping groups in Fig. 3. The corresponding values in the absence of any docked ligands are also included. The naming convention used for the BHBs is *donor residue_acceptor residue*.

**Table 1:** Docking result—docking scores for compounds with 10 best Hawkins GB/SA scores and three known inhibitors along with TMPRSS2 residues with which they interact

PubChem CID	Grid-based Score (kcal/mol)	Hawkins GB/SA Score (kcal/mol)	Hydrophobic/van der Waals Interactions	Electrostatic Interactions
102148004	− 83.58	− 60.06	Gln438, Thr459, Trp461	Val280, His296, Glu299, Lys342, Gly439, Ser441, Gly462, Ser463
5464201	− 75.38	− 59.42	Gln438, Trp461	Gly439. Ser441, Gly462
44430647	− 75.67	− 58.96	Thr459, Trp461	Val280, His296, Lys342, Ser441, Gly462
445400	− 69.61	− 57.61	Val280	Thr393, Ser436, Cys437, Gly439, Ser441, Gly462, Cys465
9939783	− 75.10	− 57.15	Lys342, Trp461	His296, Asp435, Ser436, Gly439, Ser441, Trp461
24749175	− 75.08	− 56.30	Gln438, Trp461	His296, Lys342, Asp435, Ser436, Gln438, Trp461, Gly464
52914324	− 72.10	− 55.83	Lys342, Leu419, Trp461	Lys342, Ser436, Gly439, Ser460, Ser463, Gly464
3849	− 64.94	− 55.54	Val280, Leu419, Thr459, Trp461	Gly439, Ser441, Gly462
137704649	− 63.44	− 55.23	Leu302	Val280, His296, Gly439, Ser441
146683669	− 64.91	− 55.13	Lys342, Trp461	Lys390, Asp435, Ser436, Gln438, Ser441, Gly464, Arg470

**Table 2:** Docking result—number of wrapping groups for 16 BHBs in case of apo TMPRSS2, average number of groups added for 1165 docked ligands, and groups added for the docked inhibitors camostat, nafamostat, and GBA

Backbone hydrogen bond	Wrapping groups (Apo)	ΔGroups			
		Docking average	Camostat	Nafamostat	GBA
Ala386_Gln438	11	6.51 ± 3.10	10	14	5
Trp461_Val473^a^	26	6.41 ± 2.90	5	5	5
Asp440_Cys437	12	4.21 ± 2.39	5	5	5
Gly282_Ser441	13	2.76 ± 1.79	5	4	3
Ser460_Val473	28	1.94 ± 1.50	0	0	0
Ala427_Gly472	29	1.84 ± 1.73	0	0	0
Cys297_Ala294	21	1.55 ± 2.07	1	0	0
Val280_Leu273	23	1.38 ± 1.81	4	4	0
Thr459_Gly443	13	0.88 ± 0.93	0	0	0
Gly442_Thr459	15	0.88 ± 0.93	0	0	0
Cys281_Leu273^a^	21	0.71 ± 1.02	2	3	0
Gly443_Asp440	7	0.41 ± 0.66	2	1	2
Lys342_Asp338	19	0.32 ± 0.80	0	0	0
Leu302_Glu299	22	0.27 ± 0.77	0	0	0
Val298_Ala295	29	0.13 ± 0.43	0	0	0
Gly391_Glu388	16	0.11 ± 0.51	0	0	0

^a^Double BHB where each residue acts as a donor and an acceptor

**Fig. 4:**
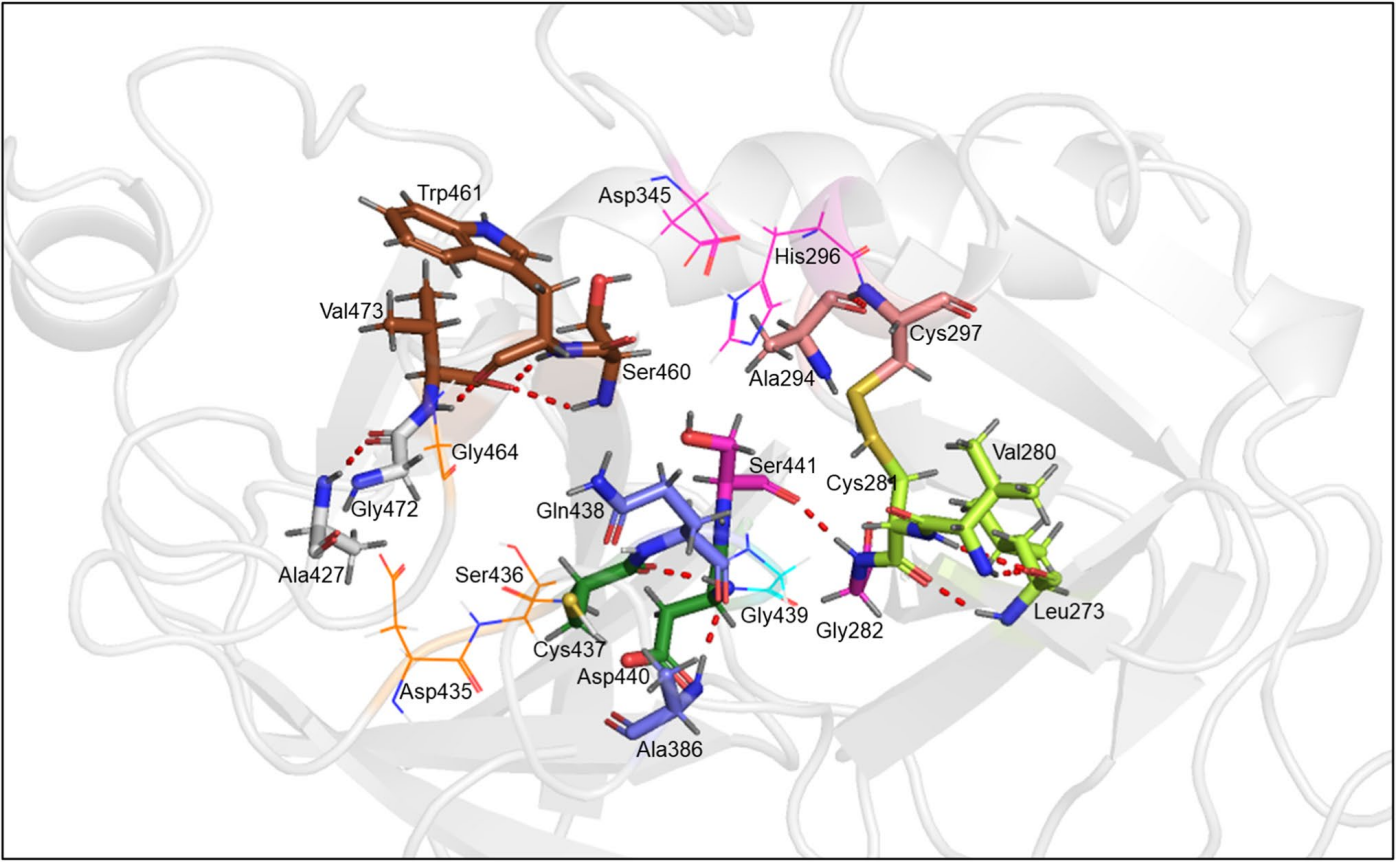
Backbone hydrogen bonds surrounding the binding pocket of TMPRSS2 shown as dotted red lines between colored residues: Ala386_Gln438 (purple), Ala427_Gly472 (gray), Asp440_Cys437 (forest green), Cys281_Leu273 and Val280_Leu273 (lime green), Cys297_Ala294 (salmon), Gly282_Ser441 (magenta), and Ser460_Val473 and the two BHBs between Trp461 and Val473 (brown), where the colors are those of their respective carbon atoms. His296 and Asp345 of the catalytic triad (magenta), Asp435, Ser436, and Gly464 of the S1 pocket (orange), and Gly439 of the oxyanion pocket (cyan) are depicted as lines

Figure 4 shows the important regions of the TMPRSS2 binding pocket—the catalytic triad (magenta) Asp345, His296, and Ser441, the S1 site (orange) essential for substrate binding which includes the residues Asp435 and Ser436, and Gly464, and the oxyanion hole formed by the Gly439 and Ser441 backbone NH groups depicted as thin lines. It also shows some nearby BHBs as red dotted lines between residues depicted as sticks of different colors. From Table 2, each of Ala386_Gln438 and Trp461_Val473 on average gain more than 6 wrapping groups as a result of docked ligands, the highest out of all BHBs. The former is located close to the oxyanion hole, while the latter lies across the binding pocket from it. An average of 4.21 and 2.76 wrapping groups are added to the BHBs Asp440_Cys437 adjacent to the oxyanion hole and Gly282_Ser441 adjacent to the catalytic triad, respectively. Besides these, Ser460_Val473, Ala427_Gly472, Cys297_Ala294, and Val280_Leu273 gain an average of 1.94, 1.84, 1.55, and 1.38 groups, respectively.

The descriptor values for most of these BHBs are strongly correlated with Δ*G*_sa_, as discussed in the following section, suggesting a relationship between wrapping and Δ*G*_sa_. From Fig. 3, the BHBs Ala386_Gln438, Asp440_Cys437, Gly282_Ser441, and Gly443_Asp440 have fewer wrapping groups in the apo case compared to other BHBs. They involve the residues Cys437, Gln438, Asp440, and Ser441, which all lie close to one another Additionally, the first three of these BHBs on average gain a large number of wrapping groups from docking and hence, may constitute a desolvation hotspot.

**Fig. 5:**
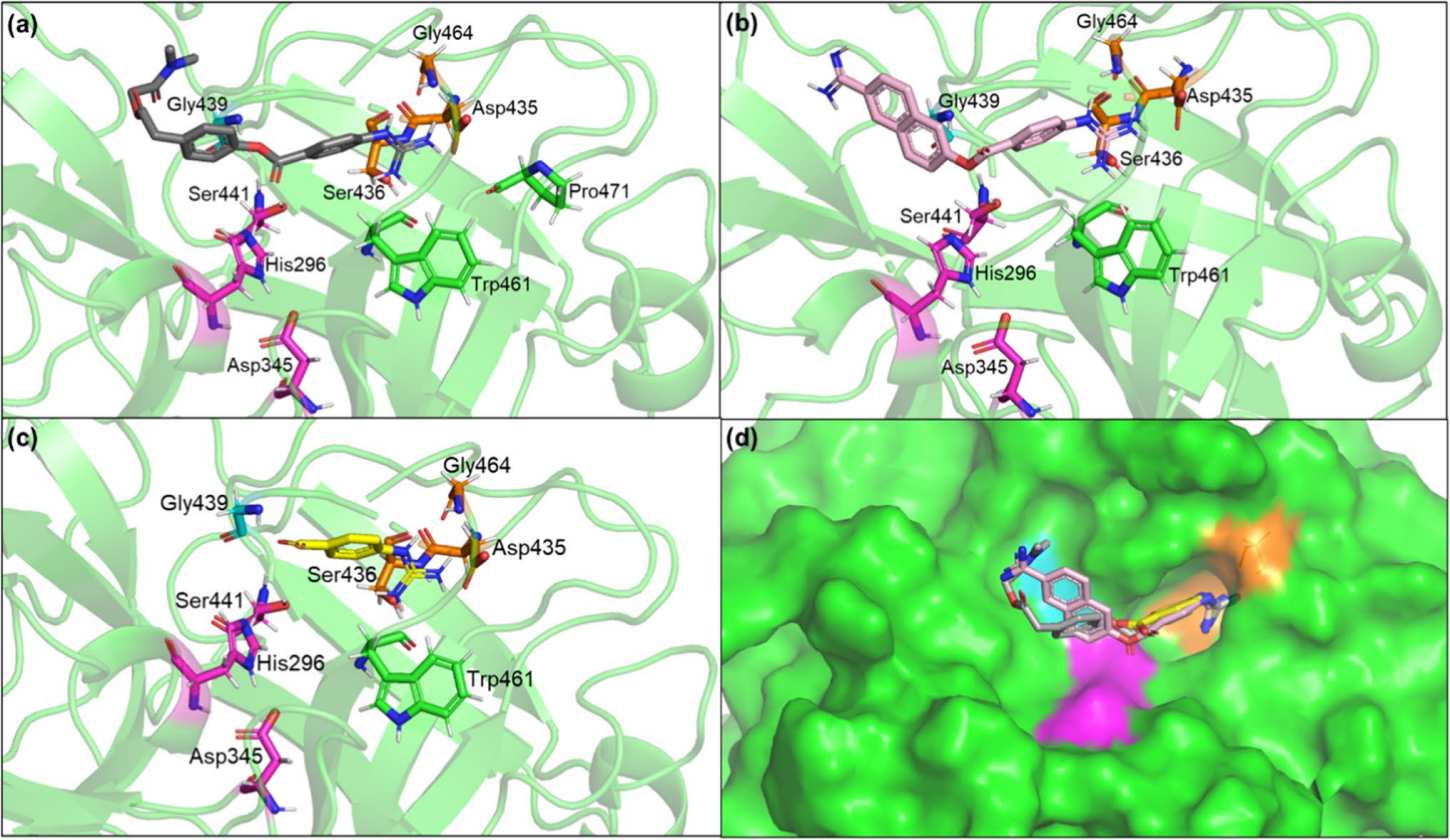
Docking poses of camostat in gray (**a**), nafamostat in light pink (**b**), and GBA in yellow (**c**). All three poses are shown in (**d**) along with surface representation of TMPRSS2. The catalytic triad comprising His296, Asp345, and Ser441 is shown in magenta. Important residues Asp435, Ser436, and Gly464 of the S1 pocket are shown in orange. Gly439 is shown in teal

Along with the inhibitor database, docking of the known TMPRSS2 inhibitors camostat, nafamostat, and GBA was also carried out and their docking poses were found to be similar to those reported previously (Hempel et al. 2021; Hoffmann et al. 2021a). As shown in Fig. 5, the positively charged guanidinium head for all three lies in the S1 pocket making electrostatic interactions via salt bridge or hydrogen bonding. This involves two or more of the S1 residues shown in Fig. 4 and Trp461, and in the case of camostat, also Pro471. The ester group in camostat and nafamostat, which is hydrolyzed to form the covalent complex, interacts with the oxyanion hole. It accepts hydrogen bonds from both, Gly439 and Ser441, in the former’s case and from only Ser441 in the case of the latter. The carboxyl group in GBA forms hydrogen bonds with both. These interactions likely stabilize the inhibitors in the binding pocket and enable the formation of a covalent bond with TMPRSS2.

Camostat, nafamostat, and GBA contribute 10, 14, and 5 wrapping groups to Ala386_Gln438, respectively, and each of them contributes 5 to both, Asp440_Cys437 and Trp461_Val473, which is in accordance with the high average values for those BHBs in Table 2. The first two BHBs and the third one lie on either side of the phenyl ring attached to the guanidinium head and all three gain 5 wrapping groups from this ring in the case of each inhibitor. In the case of the two larger inhibitors, the remaining groups for Ala386_Gln438 are from the other aromatic ring(s). Of the top four BHBs in Table 2, all three inhibitors have a lower number of wrapping groups for Trp461_Val473 than the average value from docking. This is interesting since its wrapping groups count for the apo case is much greater compared to the other three, which suggests that it may be well wrapped even without a bound inhibitor.

### Regression

**Fig. 6:**
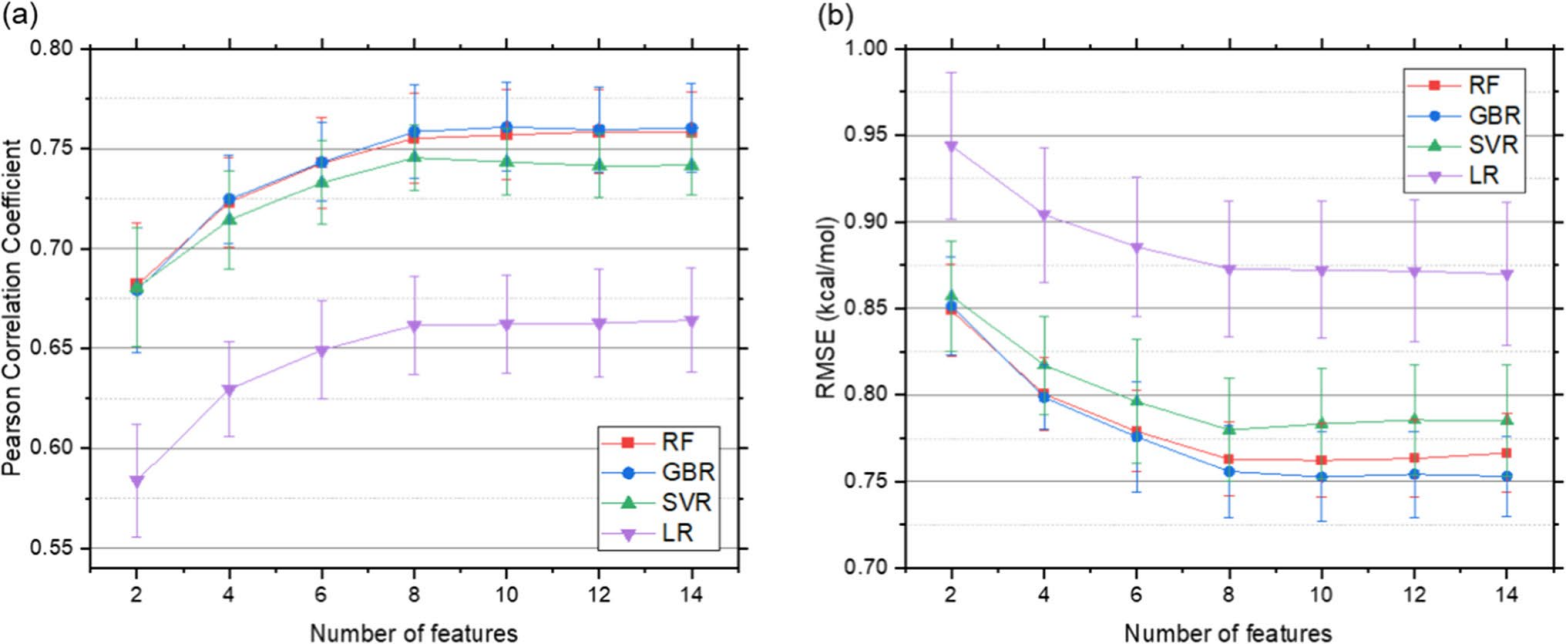
Regression performance with different number of features for Random Forest (RF), Gradient Boosting Regressor (GBR), Support Vector Regressor (SVR), and Linear Regression (LR) using two metrics: **a** Pearson correlation coefficient and **b** root mean square error (RMSE)

Machine learning was used to investigate the extent to which data encoding the wrapping patterns of BHBs could estimate Δ*G*_sa_. The dataset generated from docking poses was first subjected to feature elimination to reduce redundancies, which brings down the number of features to 14. Models to predict Δ*G*_sa_ were then created using four algorithms with varying numbers of features. The Δ*G*_sa_ term obtained from docking for all ligands ranges from − 8.66 kcal/mol to − 2.42 kcal/mol and is 10–30% of the total score for 1118 out of the 1165 ligands. The hyperparameters were selected using cross-validation by searching a grid of possible values. The selected values are provided in Online Resource 3. The performance of the regression models was evaluated using the Pearson correlation coefficient (PCC) and root mean squared error (RMSE) for predictions from ten different random train-test splits and the average values are given in Online Resource 4 and Fig. 6.

The performance with respect to both metrics for all algorithms improves with the increasing number of features until eight, beyond which additional features show little effect. For eight features, predictions from the RF, GBR, SVR, and LR had mean PCC values of 0.76, 0.76, 0.74, and 0.66, with corresponding mean RMSE values in kcal/mol of 0.76, 0.76, 0.78, and 0.87, respectively. The corresponding scatter plots between the predicted and actual Δ*G*_*sa*_ for the ten train-test splits is provided in Online Resource 5. As seen from Fig. 6, in most cases RF, GBR, and SVR all perform comparably with each of their means lying within one standard deviation of the mean for the other two. LR shows the poorest performance in all cases. The results indicate that Δ*G*_sa_ is influenced by wrapping around only a few important BHBs.

The moderate performance of the models is to be expected considering the simplicity of the wrapping descriptors. Additionally, there are no ligand descriptors in the feature set, and hence the model is not exposed to any explicit ligand properties, likely hampering its performance. The regression models are not meant for predictions anyway, but rather to verify the descriptor-target variable relationship and help identify regions in the protein that could benefit from additional wrapping groups. Nonetheless, it may be possible to build more robust models by including other descriptors along with the wrapping.

**Fig. 7:**
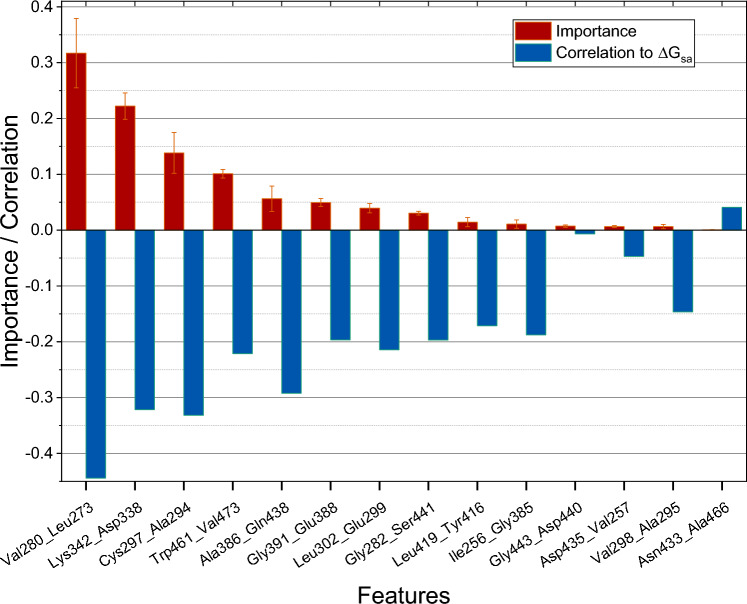
Mean feature importance calculated using gradient boosting regressor and correlation of features to Δ*G*_*sa*_

The mean importance calculated using a GBR of the various features along with their correlation to Δ*G*_sa_ is shown in Fig. 7 and Online Resource 6. Features with the three highest importance values are Val280_Leu273 (0.32), Lys342_Asp338 (0.22), and Cys297_Ala294 (0.14), with corresponding correlations to Δ*G*_sa_ of − 0.44, − 0.32, and − 0.33, respectively. Nearly all features with high importance are also strongly correlated with Δ*G*_sa_. suggesting the importance of wrapping for these BHBs. Despite its strong correlation, the feature importance of Ala386_Gln438 is relatively low. This could be because it is highly correlated to Gly282_Ser441 and Asp440_Cys437 with correlation coefficients of 0.59 and 0.50, respectively, reducing its influence on model performance. In this analysis, feature importance may be a misleading or incomplete indicator to interpret the physical effects of inhibitor binding on BHBs since features that play an important role in the binding mechanism may have been dropped during feature elimination.

### Principal component analysis

**Fig. 8:**
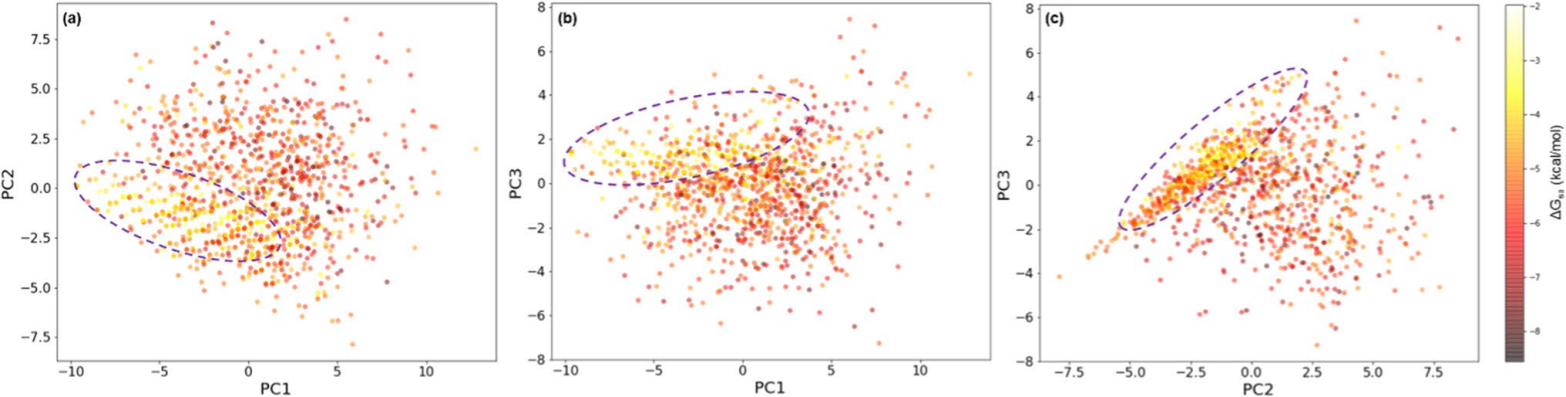
Scatter plot of data in terms of the first three principal components: **a** PC2 vs PC1, **b** PC3 vs PC1, and **c** PC3 vs PC2

To further ascertain the relationship between the wrapping of BHBs and Δ*G*_sa_ a principal component analysis of the complete dataset with 14 features was carried out. Figure 8 shows scatter plots of the data in terms of the first three principal components. The different shades of the scatter points represent different values of Δ*G*_sa_ as shown on the colormap. Distinct trends in the plot signify an underlying relationship between the features and the target variable. Most points with smaller absolute values of Δ*G*_sa_ are seen in a discernable cluster which is visible as the region enclosed by dashed ovals, while points with larger values are spread over a greater area. This suggests that the wrapping for various BHBs is similar in complexes with smaller absolute Δ*G*_sa_ and is notably different from the remaining complexes, which may be useful in identifying inhibitors that provide insufficient wrapping.

**Fig. 9:**
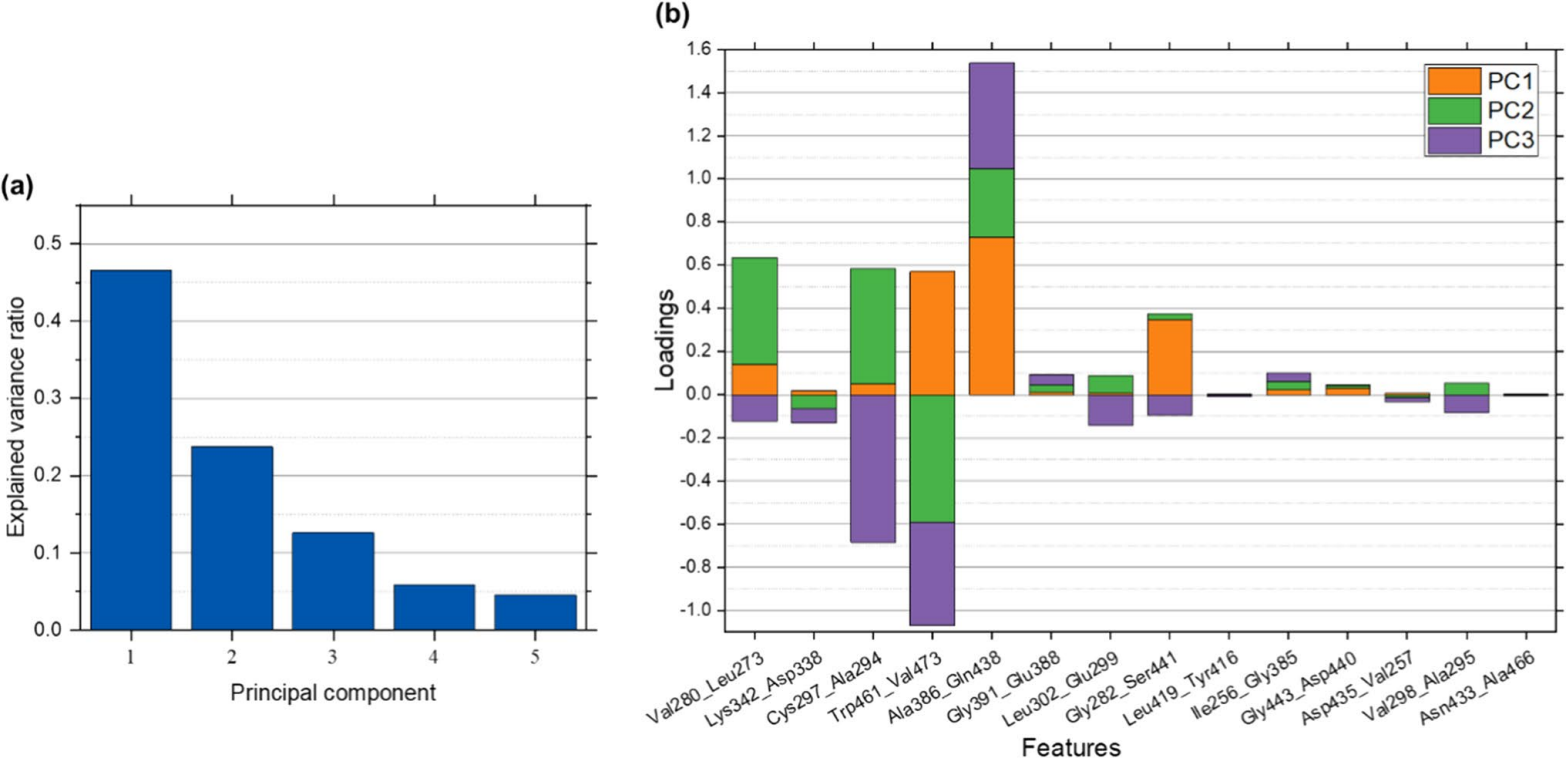
Results from PCA: **a** explained variance ratios for first five principal components; **b** Feature loadings of first three principal components

Figure 9a shows the explained variance ratios for the first 5 principal components (PCs). The first, first three, and first five PCs account for 0.47, 0.83, and 0.93 percent of the explained variance, respectively. Hence, it may be possible to further simplify the regression models by replacing the features with PCs. Loadings, which are the coefficients in features space, for the first three PCs in (a) are shown in Fig. 9b. These data are also tabulated in Online Resource 7. The PC loading corresponding to a particular feature is a measure of the influence that that feature has on the PC. The features Val280_Leu273, Cys297_Ala294, Trp461_Val473, and Ala386_Gln438 show the largest combined loading magnitudes for the first three PCs. These features also have the highest importance values in Fig. 7 and have correlation coefficients with absolute values greater than 0.2 with respect to Δ*G*_sa_ suggesting that their wrapping is likely important for Δ*G*_sa_.

### MD simulation

**Fig. 10:**
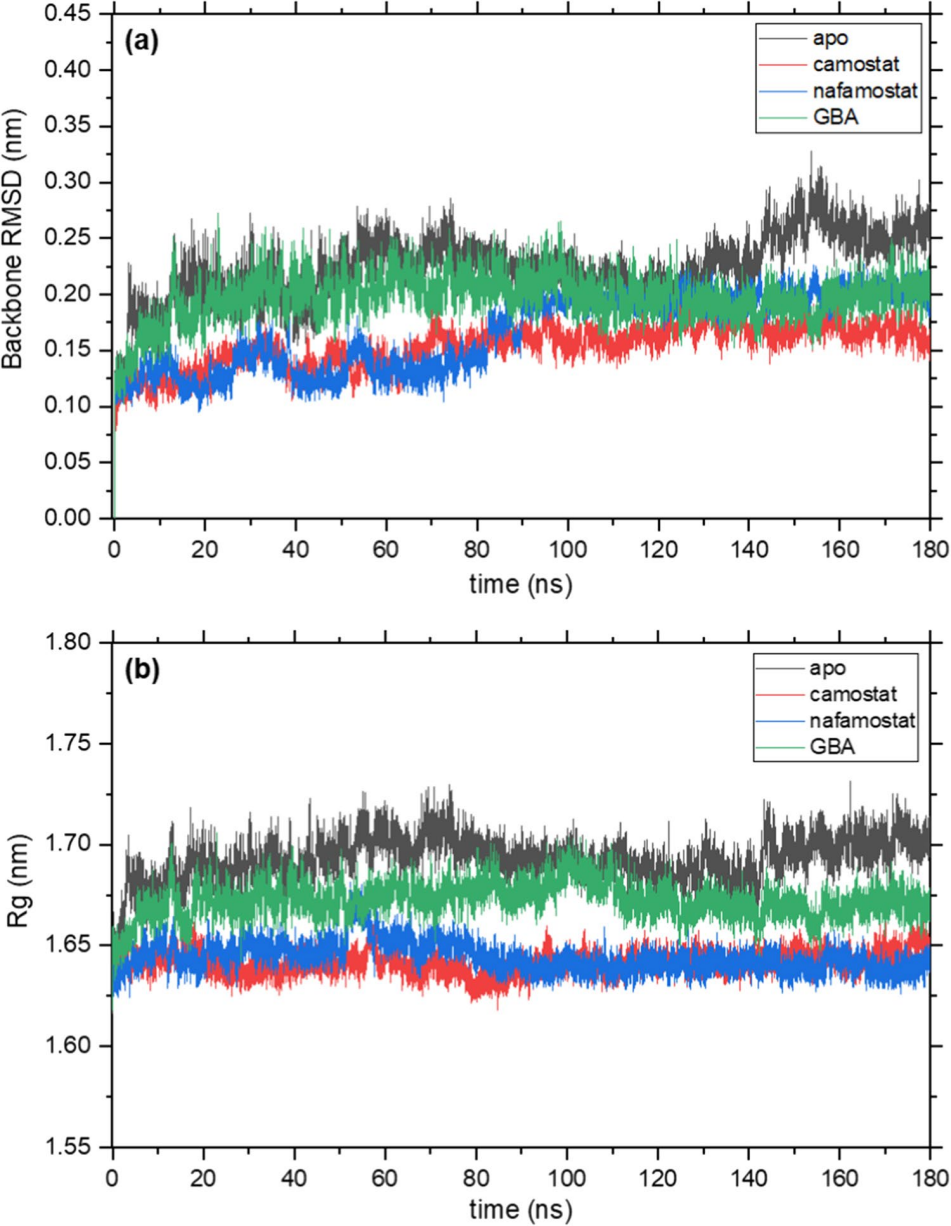
**a** Root mean square deviation (RMSD) and **b** radius of gyration (*R*_g_) of the TMPRSS2 backbone for simulations of apo protease, and its complex with camostat, nafamostat, and GBA

The effect of the inhibitors camostat, nafamostat, and GBA on the BHBs of TMPRSS2 was studied using molecular dynamics (MD). For this, 180 ns simulations of their complexes, as well as uncomplexed TMPRSS2 were carried out. The root mean square deviation (RMSD) of a protein and radius of gyration (*R*_g_), which quantifies its compactness, are parameters typically used to monitor the stability and conformational changes during simulations. The time evolution of RMSD of the TMPRSS2 backbone with respect to the structure at time zero is shown in Fig. 10a for the four simulations and the corresponding backbone *R*_g_ is shown in Fig. 10b. All RMSD curves flatten out within the first 20 ns of simulation, beyond which they undergo only small fluctuations. The average RMSD and *R*_g_ values over the 180 ns are given in Table 3. The apo protein simulation has the highest average RMSD, followed by GBA, while camostat and nafamostat have lower and comparable RMSDs. The instantaneous fluctuations in RMSD are also larger in the apo and GBA simulations compared to the other two. The *R*_g_ plot follows a similar trend, with the average values and fluctuations for the apo protein and the GBA complex being larger than those for the other two complexes. Hence, the binding of these inhibitors appears to improve the stability of TMPRSS2.

**Table 3:** Average root mean square deviation (RMSD) and radius of gyration (*R*_g_) of TMPRSS2 backbone for four 180 ns MD simulations

	Apo	Camostat	Nafamostat	GBA
RMSD (nm)	0.22 ± 0.03	0.15 ± 0.02	0.17 ± 0.03	0.2 ± 0.02
*R*_g_ (nm)	1.69 ± 0.01	1.64 ± 0.01	1.64 ± 0.01	1.67 ± 0.01

**Fig. 11:**
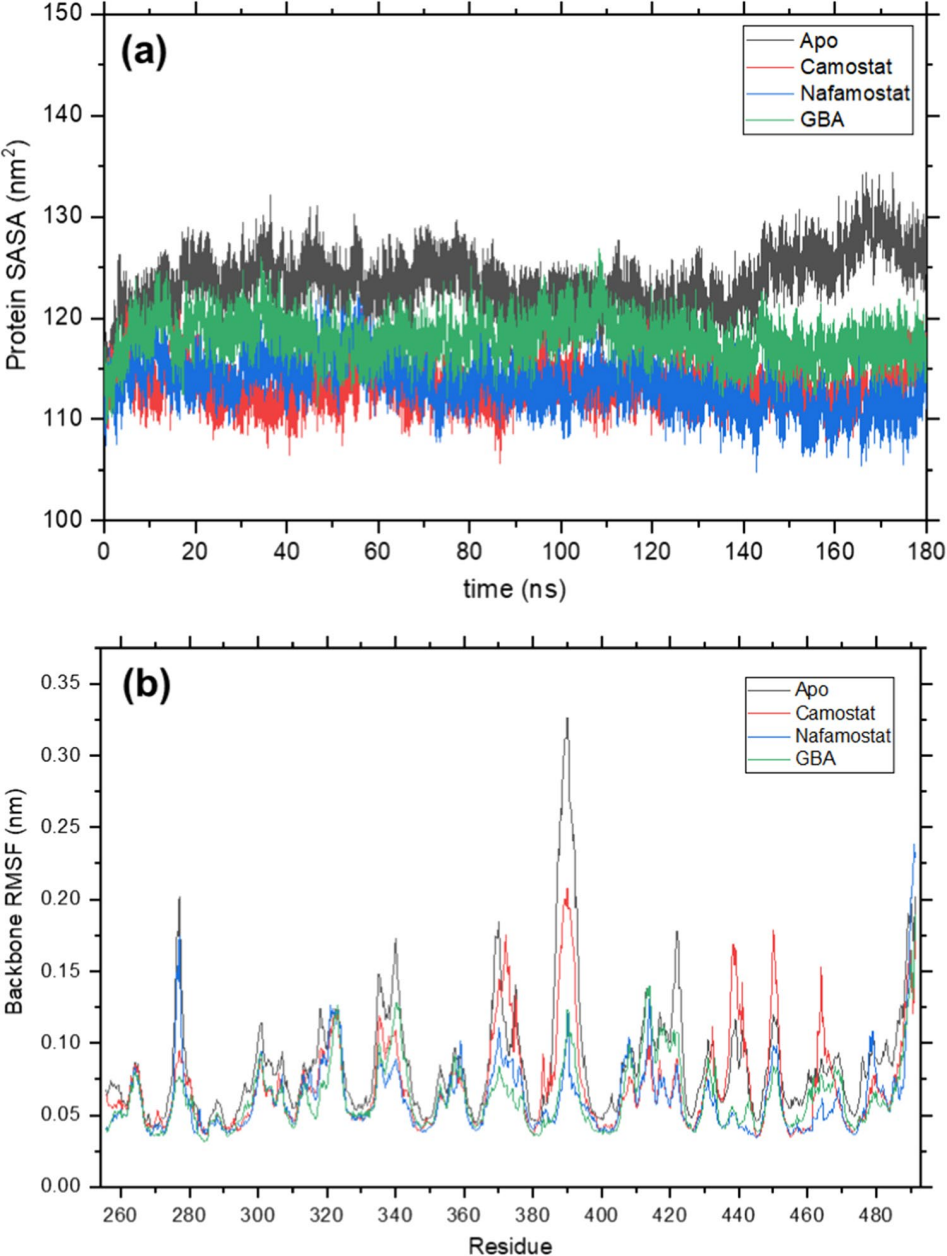
**a** Protein solvent accessible surface area (SASA) and **b** backbone root mean square fluctuation (RMSF) for simulations of apo TMPRSS2, and its complex with camostat, nafamostat, and GBA

The solvent accessible surface area (SASA) of TMPRSS2 throughout the simulation and the root mean square fluctuation (RMSF) of its backbone, calculated from the final 50 ns, are shown in Fig. 11. Proteins tend to minimize the exposure of their non-polar residues to water due to the hydrophobic effect (Schmidtke et al. 2011). Hence, the SASA is also an important quantity to assess structural stability in proteins. As would be expected, the apo protein, which does not have a bound inhibitor, shows a higher SASA compared to the complexes in Fig. 11a. This is also consistent with its larger *R*_g_, since a less compact protein structure leaves a greater area exposed to water. The binding of inhibitors camostat and nafamostat results in greater desolvation of the protein, while the SASA for the GBA complex is higher due to its smaller size. The RMSF measures the movement of atoms about fixed positions. The backbone RMSF, shown in Fig. 11b, is highest for most residues in the case of the apo protein. However, the backbone atoms near residues 438, 450, and 464 for the camostat complex show a larger fluctuation, indicating greater movement in the presence of the inhibitor.

For the complexes, the guanidine group of the inhibitors interacted with Asp435, Ser436, and Gly464 in the S1 pocket via salt bridge or hydrogen bonding, which ensured that that end was held in place. This association appeared stronger for nafamostat than camostat and strongest for GBA. For the two larger drugs, the other end with the dimethyl amide or amidine moved more freely. Both of these inhibit TMPRSS2 by covalently binding to it and this preceding complex is known to be metastable (Hempel et al. 2021), which may explain the movement. The ester group in these inhibitors and the carboxyl group in GBA interacted electrostatically with His296 and Ser441 of the catalytic triad.

**Fig. 12:**
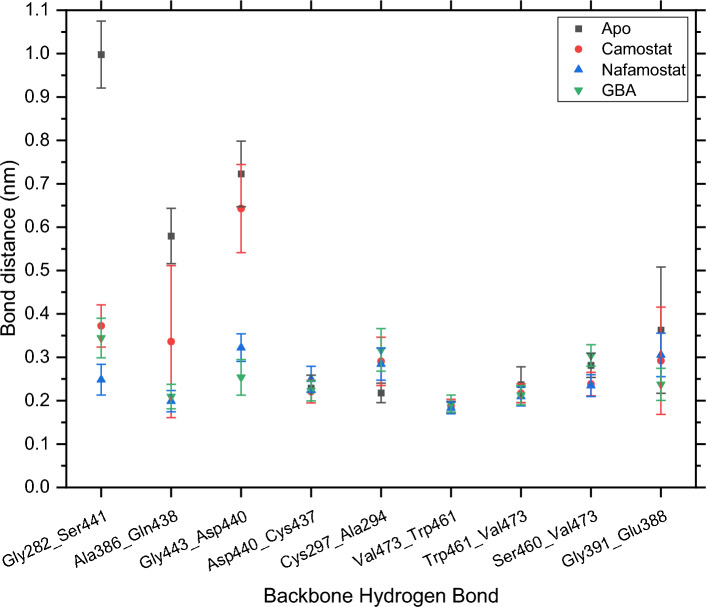
Average distance in nm between donor hydrogen and acceptor from the last 50 ns of the MD simulations

The analysis of important BHBs in all four simulations is based on the last 50 ns of each trajectory. The calculation of the average number of wrapping groups was performed using 1000 evenly spaced frames drawn from this portion of the simulation. The BHBs with the largest increase in wrapping due to the presence of inhibitors are reported in Table 4. Frames where the distance between a donor hydrogen and acceptor oxygen exceeds 0.35 nm were not considered in calculating the wrapping groups of that BHB. The number of frames out of 1000 used to calculate the average is given in parentheses. A large value represents a stable BHB, while a small value represents a weakly associated BHB that breaks and forms intermittently or seldom forms. The corresponding average distance between donor hydrogens and acceptor oxygens during the 50 ns is shown in Fig. 12 and Online Resource 8.

**Table 4:** Average number of wrapping groups for BHBs and number of MD frames used to calculate the average

Backbone hydrogen bond	Apo	Camostat	Nafamostat	GBA
Gly282_Ser441	–	15.03 ± 1.94 (36)	12.17 ± 1.59 (764)	14.69 ± 1.55 (52)
Ala386_Gln438	–	9.63 ± 0.97 (595)	15 ± 1.66 (971)	13.18 ± 1.06 (920)
Gly443_Asp440	–	–	12.02 ± 1.19 (853)	10.81 ± 1.72 (698)
Asp440_Cys437	10.16 ± 0.85 (891)	12.4 ± 1.26 (908)	13.95 ± 1.1 (607)	17.33 ± 0.93 (928)
Cys297_Ala294	20.34 ± 1.56 (949)	26.66 ± 1.76 (287)	21.68 ± 1.81 (310)	20.36 ± 1.46 (176)
Val473_Trp461	22.88 ± 1.52 (1000)	26.48 ± 1.64 (999)	28.07 ± 1.21 (999)	28.81 ± 2.11 (992)
Trp461_Val473	22.92 ± 1.53 (815)	26.48 ± 1.64 (974)	28.05 ± 1.21 (972)	28.78 ± 2.11 (967)
Ser460_Val473	25.98 ± 1.84 (634)	28.44 ± 1.92 (869)	28.64 ± 1.66 (794)	30.54 ± 2.55 (151)
Gly391_Glu388	11.78 ± 1.06 (357)	14.19 ± 1.34 (610)	16.48 ± 1 (192)	15.35 ± 0.8 (697)

Similar to the results from docking, Gly282_Ser441, Ala386_Gln438, and Gly443_Asp440 show a large increase in the average number of wrapping groups in the presence of inhibitors, except for Gly443_Asp440 in the case of camostat. This is assuming their values to be 0 in the apo case, since the apo TMPRSS2 simulation had no frames where the donor H-acceptor distance was within 0.35 nm. For the uncomplexed system, starting with the crystal structure the protein initially underwent some conformational changes and as a result, the three BHBs were either unable to persist or became unstable, which is also reflected in their high average distance as seen in Fig. 12.

In comparison, Gly-282_Ser-441 was much more stable in the presence of inhibitors, considering the number of frames in which it was formed, as well as bond distance. However, it seems to favor a smaller number of wrapping groups since its bond distance is shortest for nafamostat, which contributed an average of 12.2 groups, the fewest among the three inhibitors. There was a considerable increase in the stability of Ala386_Gln438 and Gly443_Asp440 also, but to a smaller extent in the case of camostat where the average number of wrapping groups is lower—9.63 and 0, respectively—indicating insufficient wrapping. Compared to this, the average numbers for those two BHBs in the case of nafamostat are 15 and 12.02, and in the case of GBA are 13.18 and 10.81, respectively. All three BHBs have residues in the region from Cys437 to Gly443. Similar to the results from docking, BHBs in this region seem to show the largest increase in wrapping and also the largest change in bond distance.

Other BHBs with notable increases include Trp461_Val473, Val473_ Trp461, Asp440_Cys437, Ser460_Val473, Cys297_Ala294, and Gly-391_Glu-388 which all gained on average more than three wrapping groups for at least one of the complexes. Of these, Trp461_Val473, Ser460_Val473 (except in the case of GBA), and Gly-391_Glu-388 had a small decrease in the distance in the presence of the inhibitors, while Val473_ Trp461 and Asp440_Cys437 were strongly associated even in the apo state, and the presence of inhibitors did not significantly affect them. However, Cys297_Ala294 appeared weaker as evidenced by both the decrease in the number of frames in which it was formed and the larger distance for complexes. This may be due to its proximity to the catalytic triad and the consequent presence of several simultaneous interactions, which may be more favorable than desolvation. For the two BHBs between Trp461 and Val473, the difference in the number of frames in which each of them was formed is much greater in the apo protein’s case. This again points toward the greater instability of apo TMPRSS2 as compared to its complexes.

**Fig. 13:**
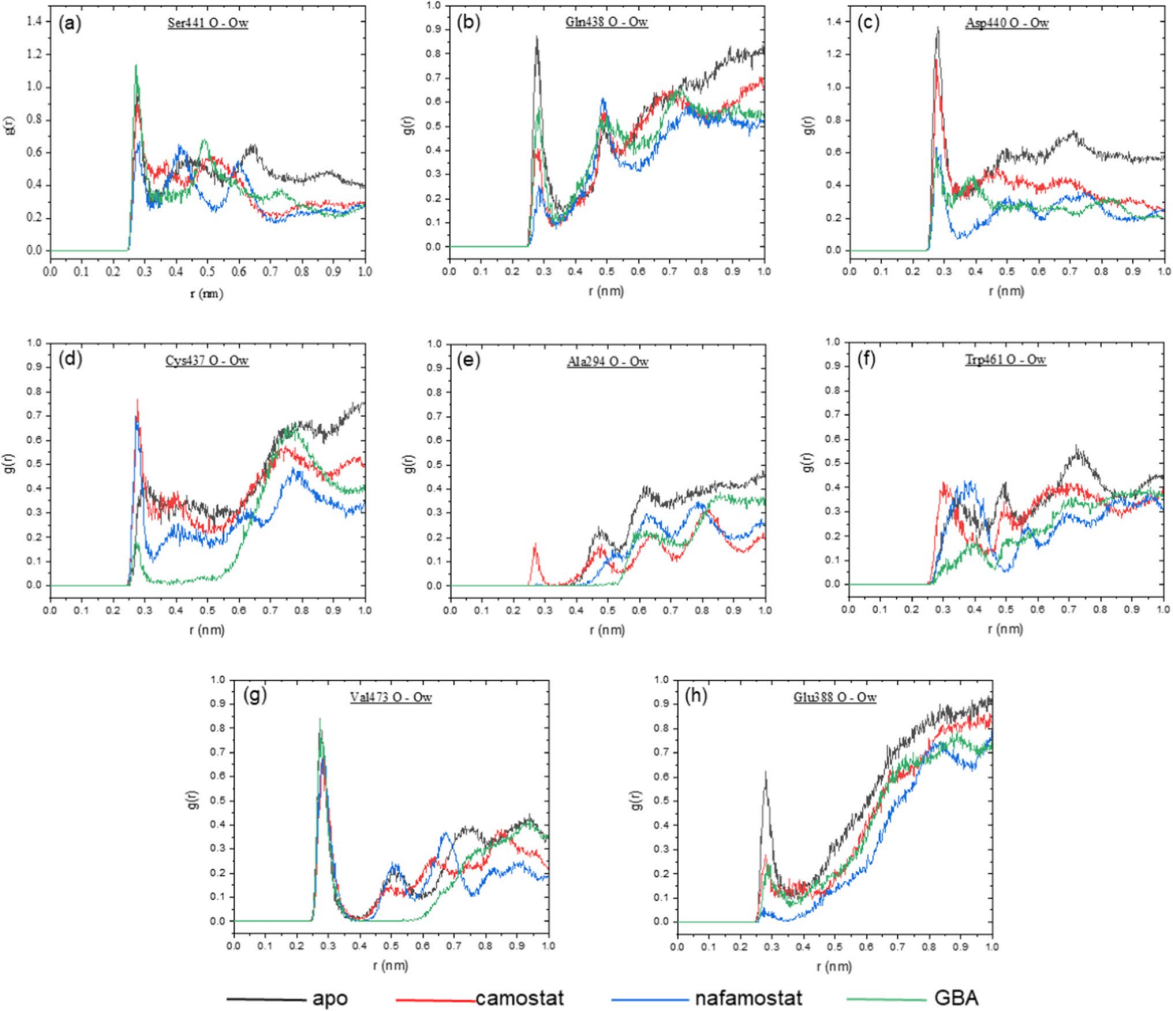
Radial distribution function of oxygen atom of water (*O*_w_) around backbone oxygen of eight BHB acceptor residues

To observe the solvent environment of various BHBs in the four simulations, the radial distribution function (RDF) of water, which describes the density of solvent around a reference atom, was calculated. Figure 13 shows the RDF for the oxygen atom of water calculated around the hydrogen bond acceptors of the BHBs in Fig. 12. The biggest disparity in RDFs between apo TMPRSS2 and its complexes is seen for Gln438 (b), Asp440 (c), Cys437 (d), and Glu388 (h), all of which except Cys437 have a distinct first peak in the apo case that represents a well-formed first solvation shell.

For Gln438, nafamostat has the shortest first peak, followed by camostat and GBA. The shortening of the first peak is a result of disruption of the solvation layer and its height could be considered an approximate measure of the extent of desolvation. For Asp440, nafamostat and GBA have comparable, moderate peaks and camostat has a tall peak, which follows from Table 4 since Gly443_Asp440 is never formed in the camostat simulation. This allows the formation of a solvation shell. Unlike the others, the RDF for Cys437 in the case of the two larger inhibitors shows the formation of a solvation shell due to inhibitor binding, while the bond distance remains roughly the same. This suggests that water molecules provide a stable bridging effect to maintain the BHB. The shorter peak in the case of GBA can be explained by the higher number of wrapping groups for it in Table 4 leading to greater desolvation. The first peak is nearly absent for Glu388 in the case of nafamostat indicating significant desolvation, while camostat and GBA have short, comparable peaks.

In the RDF around Ser441, only nafamostat shows a relatively shorter first peak, whereas it is unchanged in the case of camostat and GBA. This agrees with the observation that the nafamostat complex has the shortest average distance for Gly-282_Ser-441. The absence of a first peak for Ala294 shows that Cys297_Ala294 is well wrapped even in apo TMPRSS2, which may explain its preference for fewer wrapping groups. The small first peak for camostat is likely due to larger fluctuations as seen from its standard deviation in Fig. 12 and a result of solvation shell formation during the periods of time when the BHB was broken. This is absent in others where the BHB remained at least weakly associated throughout the simulation. A well-defined, sharp solvation peak is absent in the Trp461 RDF for all cases, while the peak is unchanged for all cases in the RDF around Val473, which is consistent with the excellent stability of the two BHBs between these residues in all simulations.

Hence, residues Gln438, Asp440, Ser441, and Glu388 all show desolvation to at least some extent, which may have contributed to the stabilization of their BHBs. Additionally, the first three of these lie in the chain from Cys437 to Gly443, which may be a desolvation hotspot. Judging from the RDFs, in general, nafamostat offers better wrapping than camostat, which could be one of the reasons for its higher effectiveness in blocking the entry of SARS-CoV-2 into host cells (Hoffmann et al. 2020a, b).

## Conclusions

This study describes the utility of a novel and easy-to-calculate descriptor based on the concept of hydrogen bond wrapping, which could be used to enhance our understanding of protein–ligand interactions*.* The descriptor quantifies the extent of wrapping around the backbone hydrogen bonds (BHBs) of a protein or its complex and can be used to identify BHBs crucial to inhibitor binding, similar to a receptor-based pharmacophore model. By modifying an inhibitor to alter the wrapping of these BHBs, one could potentially improve both, its binding affinity toward the target protein, as well as specificity.

Here, virtual screening was carried out for Transmembrane protease serine 2 (TMPRSS2) inhibitors using molecular docking with a Generalized Born surface area (GBSA) scoring function and the number of wrapping groups added to the various BHBs in the resultant poses was analyzed. The BHBs Gly-282_Ser-441, Ala386_Gln438, and Asp440_Cys437, which had a lower number of wrapping groups in the apo case compared to other BHBs, were seen to have some of the largest average increases in wrapping due to the docked inhibitors, signifying their importance in the binding process. It was also shown that a weak relationship exists between the descriptor and the surface area term Δ*G*_sa_ of the GBSA score and that the concept could possibly be used to study the change in solvent-accessible surface area due to the binding of ligands.

A similar analysis using the descriptor was also carried out for the MD trajectories of the inhibitors camostat, nafamostat, and 4-guanidinobenzoic acid (GBA) in complex with TMPRSS2. It was found that the BHBs Gly-282_Ser-441, Ala386_Gln438, and Gly443_Asp440, which were not formed in the apo case, were relatively stable in most cases in the presence of the bound inhibitors and with adequate wrapping. Interestingly, the first two of these BHBs are the same that were identified as having a large increase in wrapping from docking. In parallel with this increase in stability, it was observed using the radial distribution function of water that the well-formed solvation layer around the hydrogen bond acceptors of these BHBs in the uncomplexed TMPRSS2 is perturbed due to the inhibitors. This shows that along with the guanidine group of the inhibitors which interacts electrostatically with the S1 pocket, the groups that provide wrapping to these BHBs lying close to the binding pocket also play a role in their binding.

The descriptor offers a promising method to study the phenomenon of hydrogen bond wrapping in proteins to gain insights into the binding mechanism of inhibitors and the rational design of new inhibitors. The encouraging results from the current study and the potential of the descriptor to be developed into a powerful tool for drug design warrant further investigation of its application.

### Supplementary Information

Below is the link to the electronic supplementary material.

Supplementary file1 (XLSX 130 KB)

Supplementary file2 (PDF 576 KB)

Supplementary file3 (XLSX 21 KB)

Supplementary file4 (XLSX 18 KB)

Supplementary file5 (PDF 985 KB)

Supplementary file6 (XLSX 18 KB)

Supplementary file7 (XLSX 19 KB)

Supplementary file8 (XLSX 18 KB)

## Data Availability

Data are available upon request.
