# Supplementary material for: Inhibitor design for TMPRSS2: insights from computational analysis of its backbone hydrogen bonds using a simple descriptor
Source: Eur Biophys J. 2023 Dec 29;53(1-2):27–46. doi: 10.1007/s00249-023-01695-4 (PMC10853362; doi:10.1007/s00249-023-01695-4)

Inhibitor Discovery for TMPRSS2 and Analysis of its Backbone Hydrogen Bonds Using a Simple Descriptor

European Biophysics Journal

Suraj Ugrani (sugrani@purdue.edu)
Purdue University, West Lafayette, IN 47907, USA

Online Resource 2 Molecular structures of the ten compounds with the highest Hawkins GB/SA scores

1. PubChem CID: 102148004
   Grid-based score = -83.58 kcal/mol
   Hawkins GB/SA score = -60.06 kcal/mol

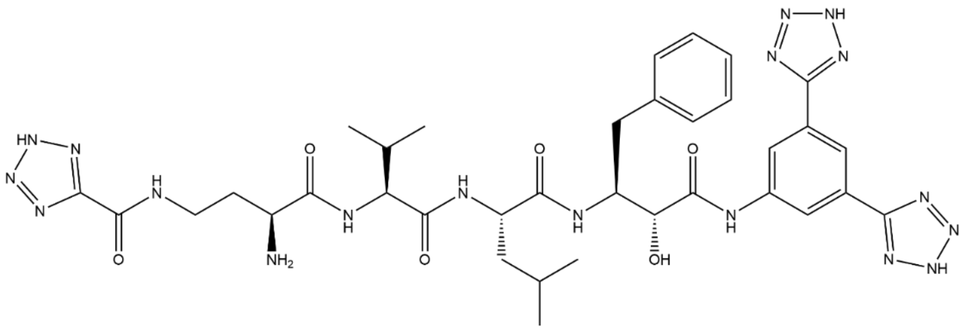

2. PubChem CID: 5464201
   Grid-based score = -75.38 kcal/mol
   Hawkins GB/SA score = -59.42 kcal/mol

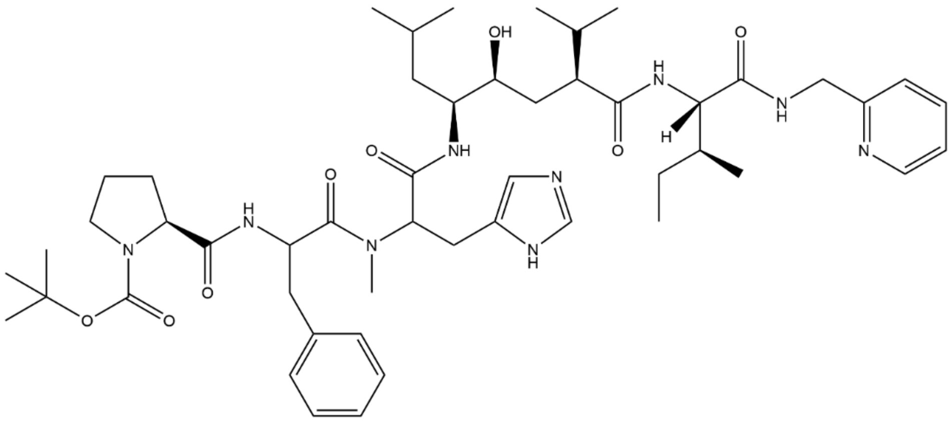

Ditekiren

3. PubChem CID: 44430647
   Grid-based score = -75.67 kcal/mol
   Hawkins GB/SA score = -58.96 kcal/mol

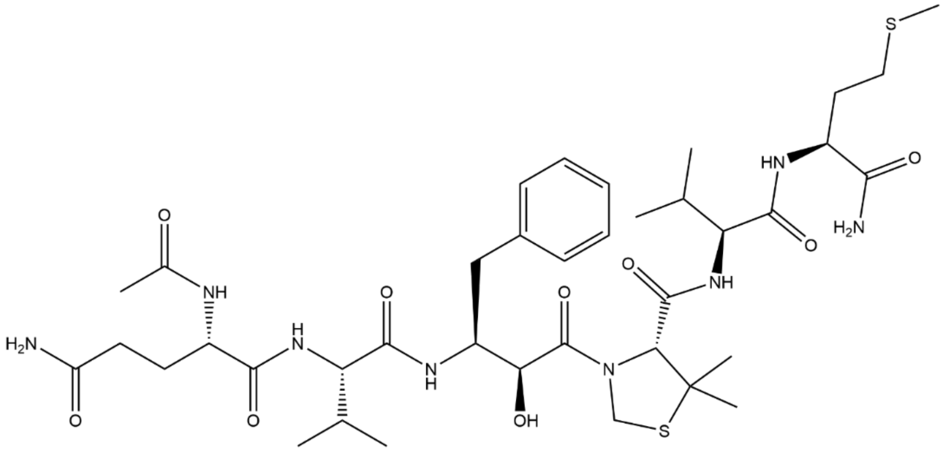

4. PubChem CID: 445400
   Grid-based score = -69.61 kcal/mol
   Hawkins GB/SA score = -57.61 kcal/mol

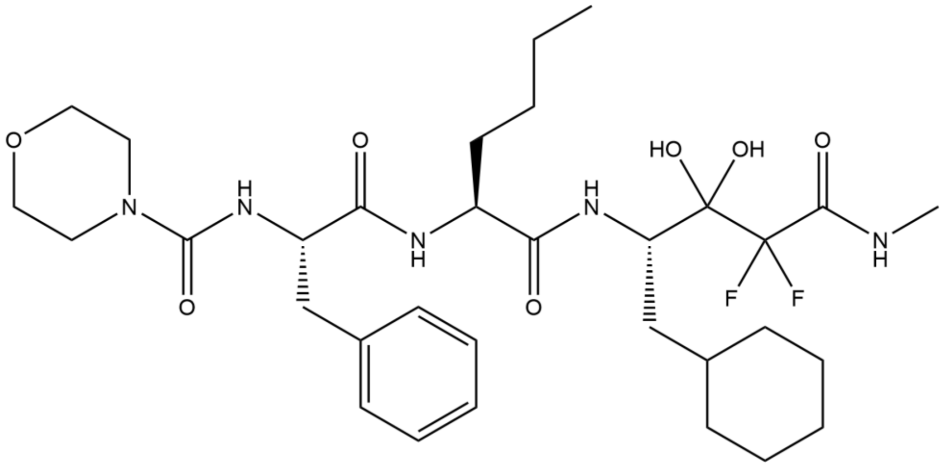

5. PubChem CID: 9939783
   Grid-based score = -75.10 kcal/mol
   Hawkins GB/SA score = -57.15 kcal/mol

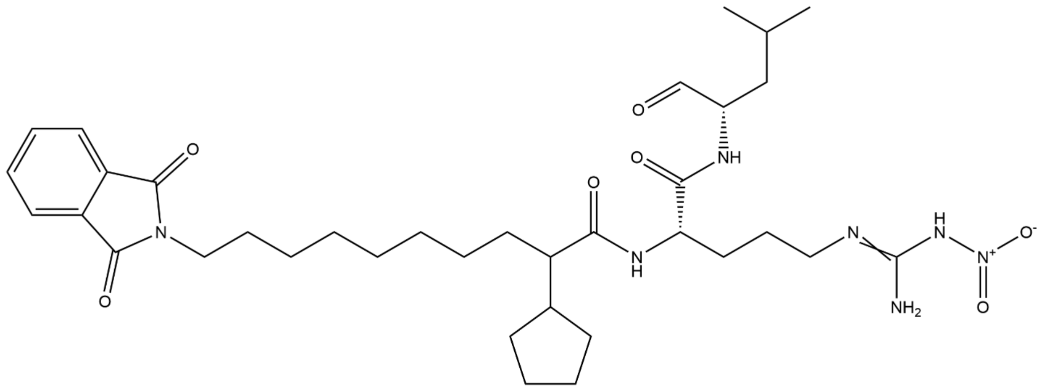

6. PubChem CID: 24749175
   Grid-based score = -75.08 kcal/mol
   Hawkins GB/SA score = -56.30 kcal/mol

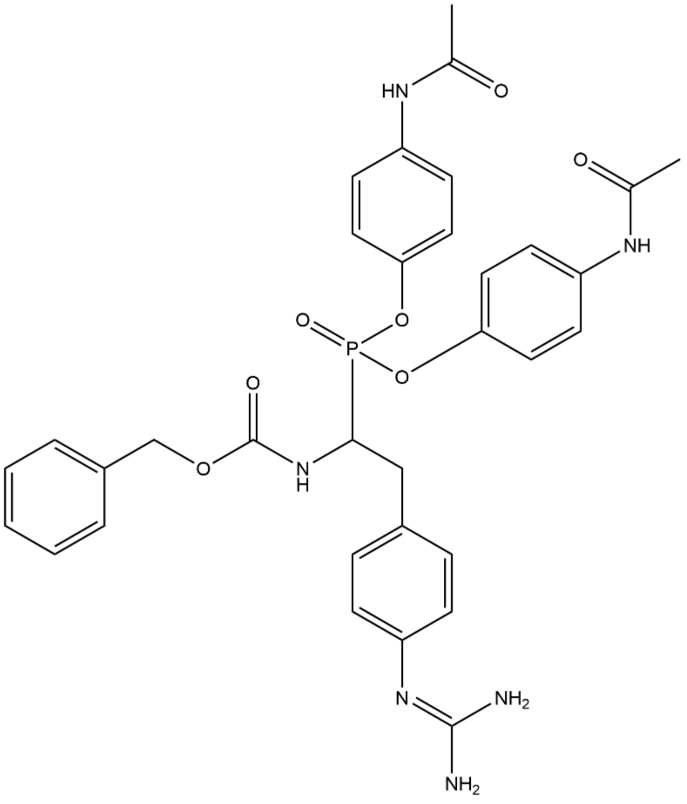

7. PubChem CID: 52914324
Grid-based score = -72.10 kcal/mol
Hawkins GB/SA score = -55.83 kcal/mol

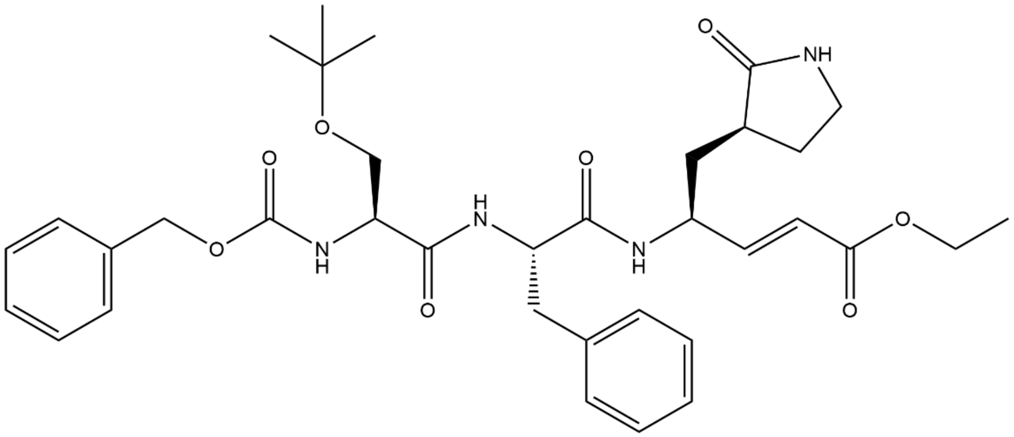

8. PubChem CID: 3849
Grid-based score = -64.94 kcal/mol
Hawkins GB/SA score = -55.54 kcal/mol

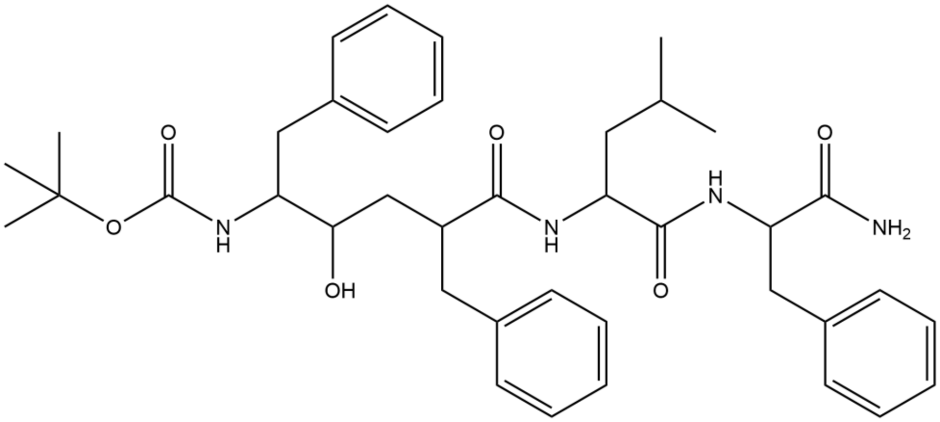

9. PubChem CID: 137704649
   Grid-based score = -63.44 kcal/mol
   Hawkins GB/SA score = -55.23 kcal/mol

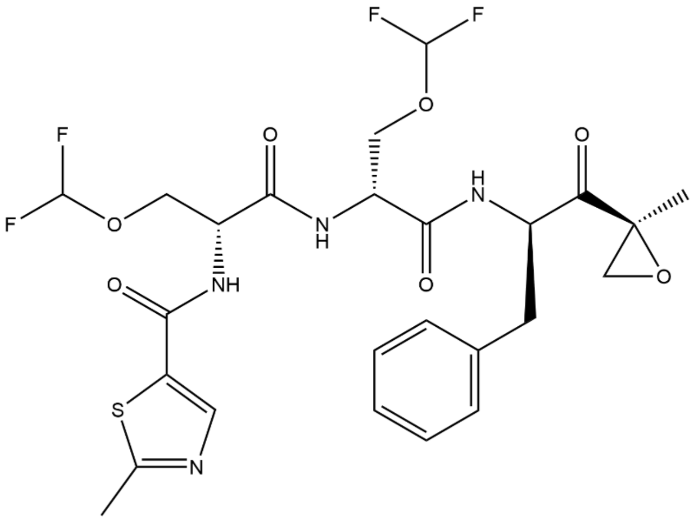

10. PubChem CID: 146683669
    Grid-based score = -64.91 kcal/mol
    Hawkins GB/SA score = -55.13 kcal/mol

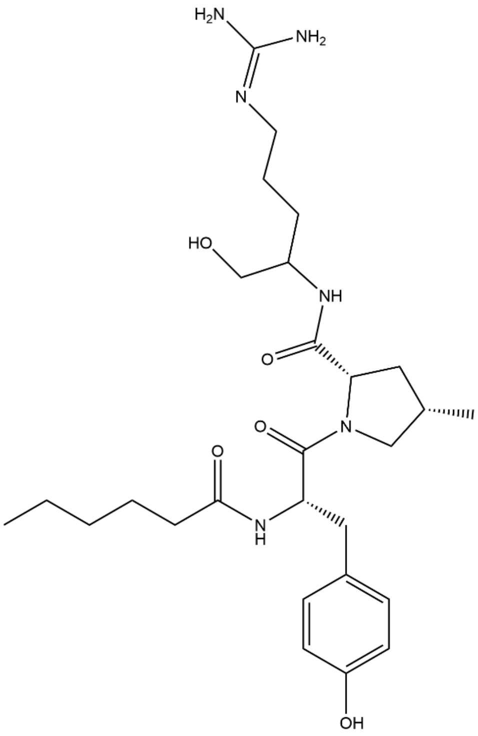

Supplement: Supplementary file 2 — Supplementary file2 (PDF 576 KB) [file 249_2023_1695_MOESM2_ESM.pdf]
