# Supplementary material for: Inhibitor design for TMPRSS2: insights from computational analysis of its backbone hydrogen bonds using a simple descriptor
Source: Eur Biophys J. 2023 Dec 29;53(1-2):27–46. doi: 10.1007/s00249-023-01695-4 (PMC10853362; doi:10.1007/s00249-023-01695-4)
Supplement: Supplementary file 5 — Supplementary file5 (PDF 985 KB) [file 249_2023_1695_MOESM5_ESM.pdf]

Inhibitor Discovery for TMPRSS2 and Analysis of its Backbone Hydrogen Bonds Using a Simple Descriptor

European Biophysics Journal

Suraj Ugrani (sugrani@purdue.edu)

Purdue University, West Lafayette, IN 47907, USA

Online Resource 5 Predicted vs actual ΔG_{sa} scatter plots for linear (LR), random forest (RFR), support vector machine (SVR) and gradient boosting (GBR) regression using 8 features for ten random train-test splits

Split 0

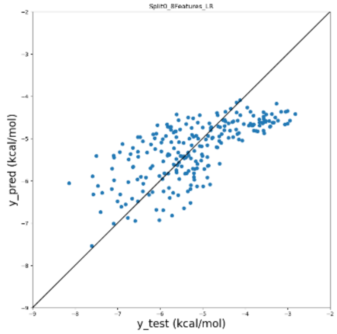

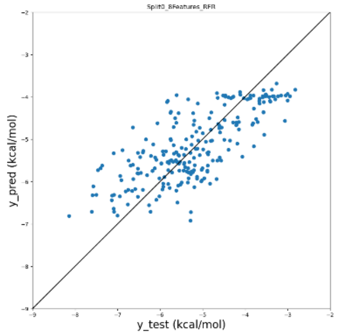

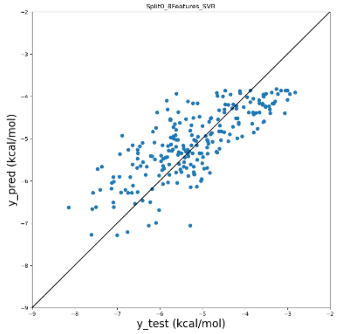

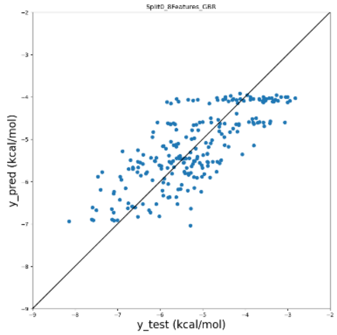

<u>Split 1</u>

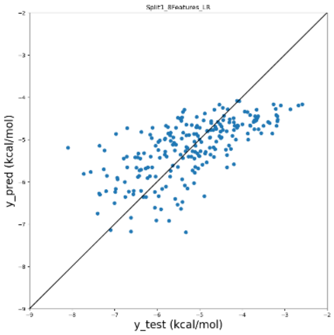

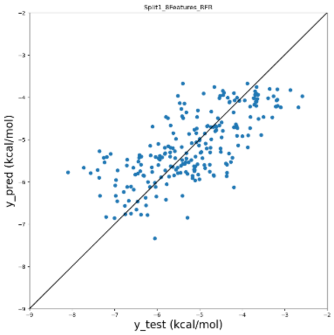

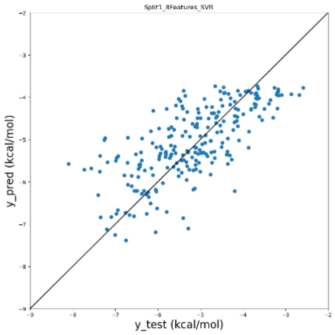

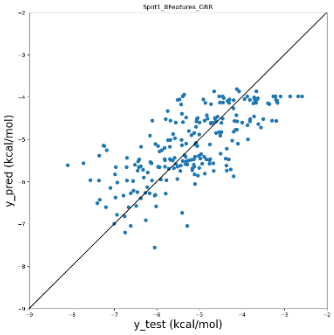

<u>Split 2</u>

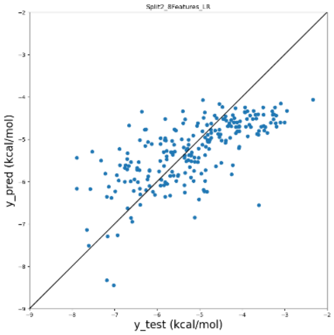

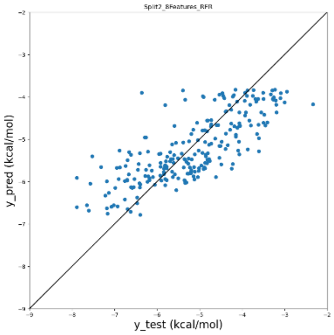

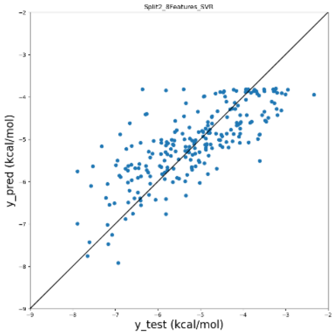

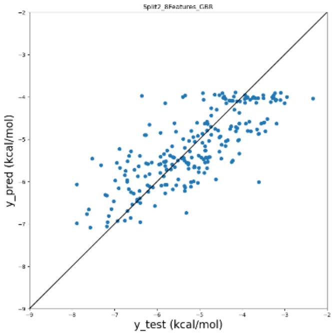

Split 3

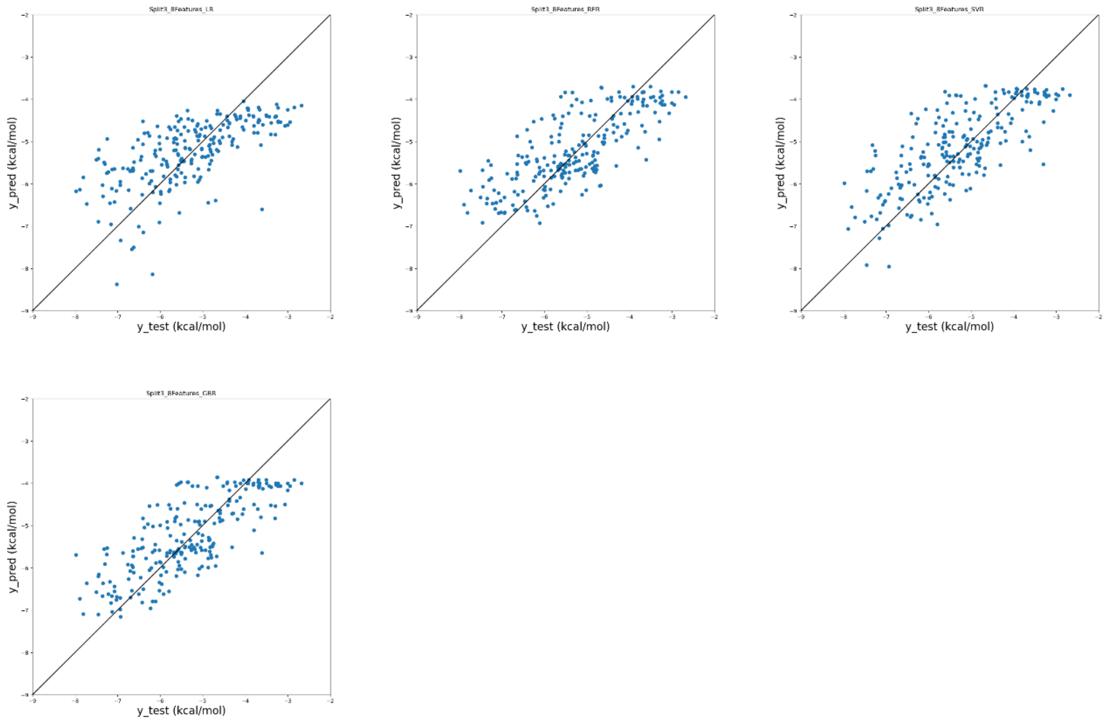

Split 4

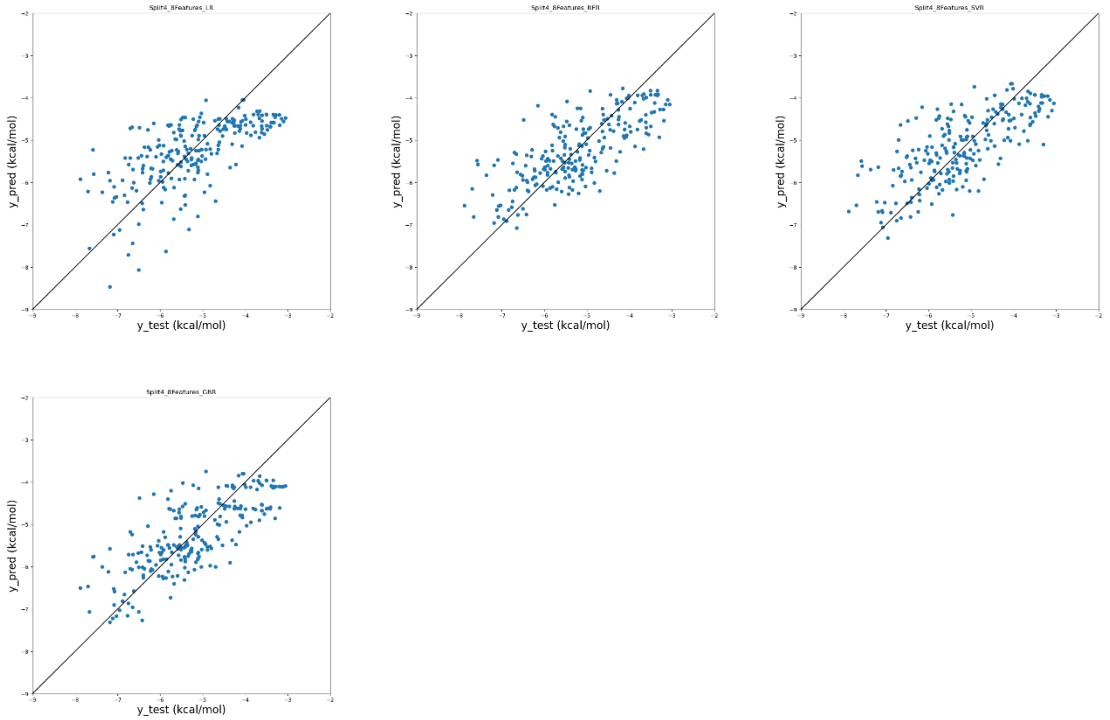

<u>Split 5</u>

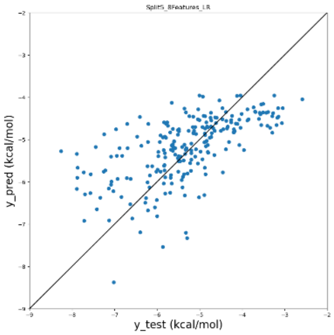

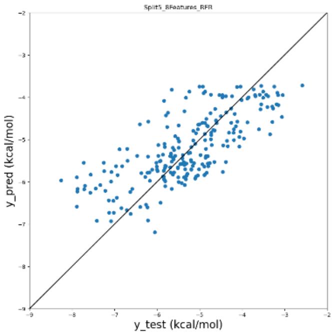

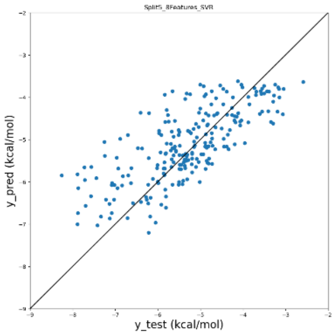

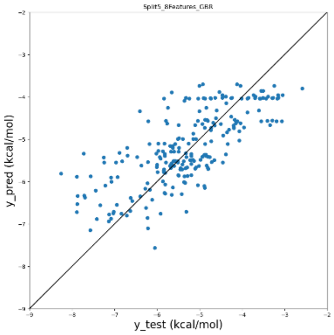

<u>Split 6</u>

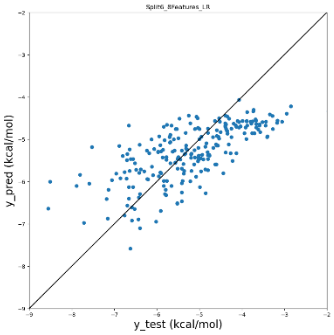

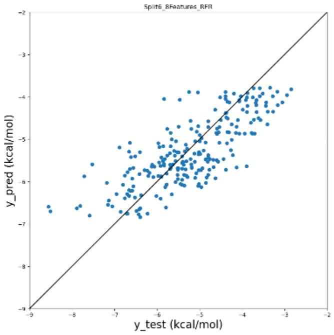

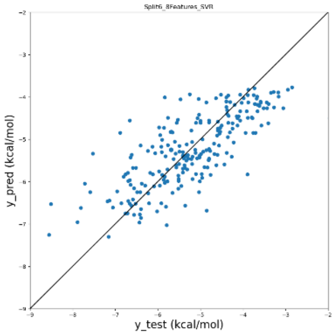

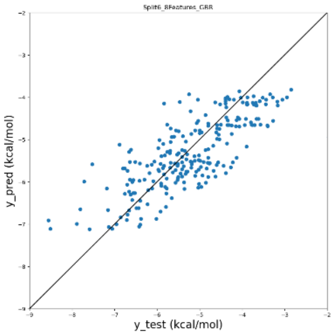

Split 7

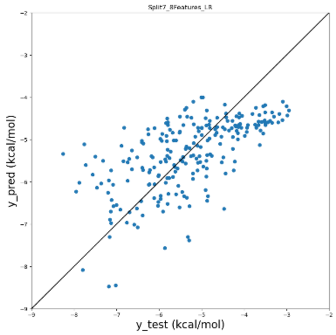

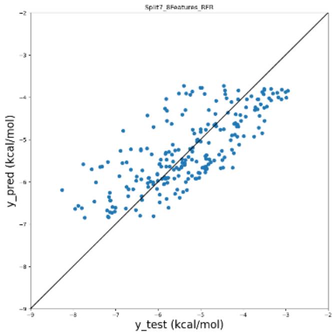

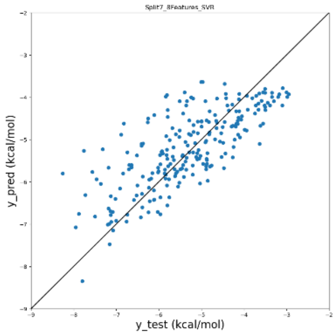

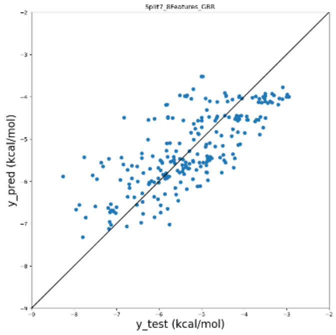

Split 8

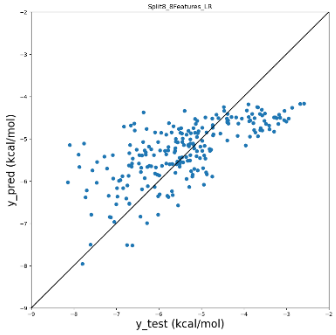

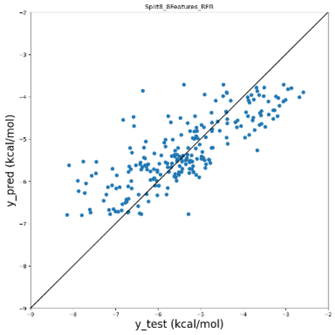

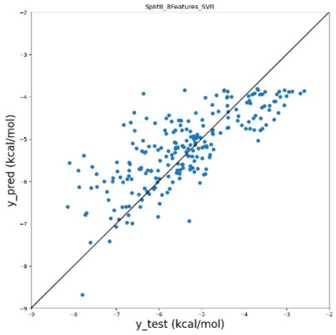

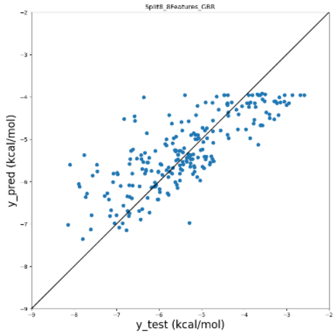

<u>Split 9</u>

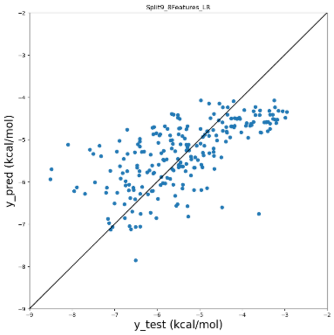
Split9_8Features_LR
y_pred (kcal/mol)
y_test (kcal/mol)

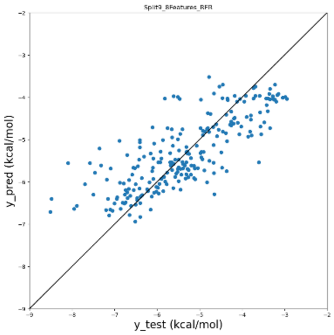
Split9_8Features_RFR
y_pred (kcal/mol)
y_test (kcal/mol)

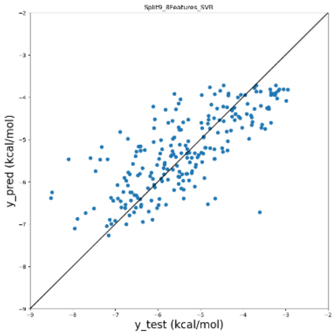
Split9_8Features_SVR
y_pred (kcal/mol)
y_test (kcal/mol)

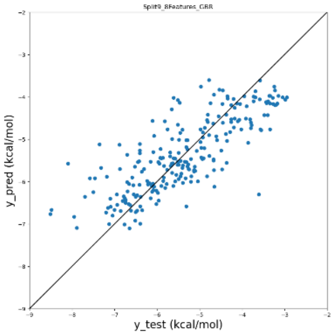
Split9_8Features_GBR
y_pred (kcal/mol)
y_test (kcal/mol)
